# Hippocampal tau oligomerization early in tau pathology coincides with a transient alteration of mitochondrial homeostasis and DNA repair in a mouse model of tauopathy

**DOI:** 10.1186/s40478-020-00896-8

**Published:** 2020-03-04

**Authors:** Jin Zheng, Mansour Akbari, Claire Schirmer, Marie-Line Reynaert, Anne Loyens, Bruno Lefebvre, Luc Buée, Deborah L. Croteau, Marie-Christine Galas, Vilhelm A. Bohr

**Affiliations:** 1 Center for Healthy Aging, Department of Cellular and Molecular Medicine, SUND, University of Copenhagen, 2200 Copenhagen N, Denmark; 2 University of Lille, Inserm, CHU Lille, UMR-S 1172 - Lille Neuroscience & Cognition, F-59000 Lille, France; 3 Laboratory of Molecular Gerontology, National Institute on Aging, NIH, 251 Bayview Blvd, Suite 100, Rm 06B133, Baltimore, MD 21224 USA

**Keywords:** Tau oligomerization, Mitochondrial homeostasis, Polymerase beta, Oxidative stress, Tauopathies

## Abstract

Insoluble intracellular aggregation of tau proteins into filaments and neurodegeneration are histopathological hallmarks of Alzheimer disease (AD) and other tauopathies. Recently, prefibrillar, soluble, oligomeric tau intermediates have emerged as relevant pathological tau species; however, the molecular mechanisms of neuronal responses to tau oligomers are not fully understood. Here, we show that hippocampal neurons in six-month-old transgenic mouse model of tauopathy, THY-Tau22, are enriched with oligomeric tau, contain elongated mitochondria, and display cellular stress, but no overt cytotoxicity compared to the control mice. The levels of several key mitochondrial proteins were markedly different between the THY-Tau22 and control mice hippocampi including the mitochondrial SIRT3, PINK1, ANT1 and the fission protein DRP1. DNA base excision repair (BER) is the primary defense system against oxidative DNA damage and it was elevated in six-month-old transgenic mice. DNA polymerase β, the key BER DNA polymerase, was enriched in the cytoplasm of hippocampal neurons in six-month-old transgenic mice and localized with and within mitochondria. Polβ also co-localized with mitochondria in human AD brains in neurons containing oligomeric tau. Most of these altered mitochondrial and DNA repair events were specific to the transgenic mice at 6 months of age and were not different from control mice at 12 months of age when tau pathology reaches its maximum and oligomeric forms of tau are no longer detectable. In summary, our data suggests that we have identified key cellular stress responses at early stages of tau pathology to preserve neuronal integrity and to promote survival. To our knowledge, this work provides the first description of multiple stress responses involving mitochondrial homeostasis and BER early during the progression of tau pathology, and represents an important advance in the etiopathogenesis of tauopathies.

## Introduction

The pathological aggregation of the microtubule-associated protein tau into filaments is a histopathological hallmark of a number of neurodegenerative diseases collectively known as tauopathies, including Alzheimer disease (AD) [5, 85]. Substantial evidence suggests that the impairment of neuronal function develops before the formation of detectable levels of insoluble tangles in tauopathy mouse models [89], and that soluble oligomeric tau intermediates, formed early during the process of tau aggregation, might be the more pathological forms of tau [38, 46, 64].

In AD brain, neurons with tau aggregation, named neurofibrillary tangles (NFTs), can survive for decades despite progressive functional impairment [59]. This suggests that neurons that develop tau pathology may activate a response mechanism to maintain cell function and to delay cell death. The molecular mechanism of this putative cellular survival response remains largely unknown.

Mitochondria are the primary organelles for the production of the cellular energy substrate ATP. They also play a central role in other key cellular processes including cytosolic calcium buffering [23], and control of apoptosis [19]. Mitochondrial dysfunction and defects cause or are central pathological components of a number of neurodegenerative diseases [25, 92].

Mitochondrial abnormalities including aberrant mitochondrial morphology have been detected early in AD patients and in experimental AD and tauopathy model systems [75, 91]. Moreover, there is interplay between mitochondrial dysfunction and tau pathology [25, 41, 57]. These observations suggest that interplay between mitochondrial dysfunction and tau pathology may initiate and drive the progression of tauopathies.

Mitochondrial DNA (mtDNA) is organized into structures called nucleoids in close association with the mitochondrial inner membrane [9]. Because the mitochondrial inner membrane is a major site of mitochondrial ROS production [10], mtDNA is expected to be constantly undergoing oxidative DNA damage such as 8-oxoG. DNA base excision repair (BER) is the prominent DNA repair pathway for the repair of oxidative DNA lesions [2] and probably the most important DNA repair pathway in human mitochondria [4, 78].

Recent reports have shown the presence of the major nuclear BER DNA polymerase, DNA polymerase β (Polβ), in mitochondria in the brain [65, 76], with a likely role in mtDNA repair. 3xTgAD mice with a 50% reduction of Polβ (3xTg AD/Polβ^+/−^), which also contain a mutated version of human tau, display exacerbated AD phenotypes with impaired cellular bioenergetics and mitochondrial dysfunction [58, 77].

Collectively, these studies suggest that tau alterations, mitochondrial abnormalities, and BER deficiencies may interact with one another to modulate response and the development of tauopathies; however, the timing and role of these processes in the early stages of tauopathy is largely unknown.

In this study, using THY-Tau22, a mouse model of tauopathy, we show that early during tau pathology, hippocampal neurons containing oligomerized tau proteins, exhibit changes in mitochondrial structure, enhanced DNA repair activity, and mitochondrial enrichment of Polβ, but no overt cytotoxicity. These events were mostly corrected to the level of wild-type littermate mice at a later stage of tau pathology when tau oligomers are no longer detectable. We conclude that early in the progression of tau pathology in hippocampal neurons when prefibrillar tau oligomers are prominent, significant changes in mitochondrial homeostasis and DNA repair take place to counter cellular stress and to promote cell survival.

## Materials and methods

### Animals

THY-Tau22 mouse (henceforth Tg), and its non-transgenic littermates (Wt) have been described before [70]. The Tg mouse was generated with a construct containing human tau46 mutated at G272V and P301S positions and expressed under the control of Thy1.2 promoter. Tau pathology begins at 3 months of age in the subiculum and CA1 subfield, two brain regions affected early in human AD, spreading from there to the hippocampal dentate gyrus and cortex in older animals [82]. At 6 months of age, Tg mice start developing spatial memory impairment and anxiety [47]. All Tg mice used in the present study were heterozygous. Non-transgenic Wt littermate mice were used as controls in all experiments. Both the THY-Tau22 and wild type littermate mice were generated from the same breeds. All mice were on C57Bl6/J background. Because no gender differences were observed in a previous report [47], data from both males and females were analyzed as a single group. In each experiment, three mice per category were analyzed (6-mo Tg, 6-mo Wt, 12-mo Tg, 12-mo Wt). All animals were kept in standard animal cages (12 h/12 h light/dark cycle, at 22 °C), with ad libitum access to food and water. The animals were maintained in compliance with institutional protocols (Comité d’éthique en expérimentation animale du Nord Pas-de-Calais, no. 0508003). All of the animal experiments were performed in compliance with and following the approval of the local Animal Ethical Committee (agreement #12787–2,015,101,320,441,671 v9 from CEEA75, Lille, France), standards for the care and use of laboratory animals, and the French and European Community rules.

### Immunofluorescence

Immunofluorescence was performed as described previously [84] using the following antibodies: TOC1 (a generous gift from Dr. Nicholas Kanaan) for prefibrillar tau oligomers [64]; AT8 (Thermo Scientific) and Tau pT212 (Life Technologies) are phospho-dependent antibodies, which are present from the early to late stages of tau pathology; anti-8oxoG (Santa Cruz Biotechnology); ANT1 (ab102032, Abcam); VDAC1 (ab14734, Abcam); OGG1 (NB100–106, Novus); Polβ (ab26343, Abcam); cleaved caspase 3 (9661 L, Cell Signaling Technology). DAPI was used as a chromatin counterstain. Hippocampal sections from Tg and Wt mice (*n* = 3 for each mouse category) were acquired using an LSM 710 confocal laser-scanning microscope (Carl Zeiss). The confocal microscope was equipped with a 488-nm Argon laser, 561-nm diode-pumped solid-state laser, and a 405-nm ultraviolet laser. The images were acquired using an oil 63X Plan-APOCHROMAT objective (1.4 NA). All recordings were performed using the appropriate sampling frequency (16 bits, 1024–1024 images, and a line average of 4). Serial sections from the three-dimensional reconstruction were acquired using Z-steps of 0.2 μm. For each section, cellular and nuclear (based on DAPI detection) fluorescence of CA1 cells were quantified using the FIDJI macro application of ImageJ (confocal microscopy platform, IMPRT, Institut de Médecine Prédictive et de Recherche Thérapeutique, Lille). Quantification corresponds to the z stack of serial confocal sections covering the entire thickness of the brain section. Cellular or nuclear fluorescence was quantified in CA1 sections from three different Tg and three different Wt littermates mice for each age (one section per mouse). The quantification shows the mean of cellular or nuclear fluorescence values per mouse.

### Cell density

Cell density was determined by counting the number of nuclei (based on DAPI staining) per surface unit in CA1 sections. Sagittal CA1 sections from 6- and 12-mo Wt and Tg mice hippocampi (*n* = 3 for each mouse category) were incubated with a solution (2.5 μg/ml in PBS) of 4′,6-diamidino-2-phenylindole (DAPI) (ThermoFischer) for 10 min at room temperature, and mounted with Vectashield/DAPI (Vector Laboratories). Nuclear DAPI fluorescence was analyzed by LSC laser scanning confocal microscopy (confocal microscopy platform, IMPRT, Institut de Médecine Prédictive et de Recherche Thérapeutique, Lille). Nuclei quantification corresponds to the z stack of serial confocal sections covering the entire thickness of the brain section. For each mouse category, the quantification shows the mean of nuclei per surface unit per mouse.

### Transmission electron microscopy

Hippocampus from Tg and Wt mice (*n* = 3 for each category) was fixed with paraformaldehyde 2%, picric acid (1.3% saturated in H_2_O) 0.1%, glutaraldehyde 1% in 0.1 M phosphate buffer for 72 h at 4 °C. Tissues were rinsed 3 times in 0.1 M phosphate buffer, post-fixed in 1% osmium tetroxide in 0.1 M phosphate buffer for 1 h at room temperature, then gradually dehydrated in ethanol, and embedded in araldite (one block par hippocampus). Thin sections (85 nm) were made with a ultracut (Leica EM UC7), contrasted with uranyl acetate 2% and lead citrate (Reynolds). Three sections per hippocampus, at the CA1 level, were observed with Zeiss EM 900 microscope and acquisition (100–200 images from different planes for each section) realized with camera gatan (Orius SC 1000). Mitochondrial area was quantified using the FIDJI macro application of ImageJ (confocal microscopy platform, IMPRT, Institut de Médecine Prédictive et de Recherche Thérapeutique, Lille) in six representative images from three different sections per block. For each mouse category, the quantification shows the mean of mitochondrial area per mouse.

### Immunoelectron microscopy

Hippocampus from Tg and Wt mice (*n* = 3 for each category) was fixed with 0.1% glutaraldehyde and 4% paraformaldehyde in 0.1 M Phosphate buffer for 24 h at 4 °C. Tissues were then rinsed in 0.1 M phosphate-buffer, dehydrated in ethanol (30 and 50%, 10 min each) at 4 °C, then 70 and 96% 10 min each at − 20 °C, and embedded in LRWhite resin (3 × 1 h − 20 °C, then overnight at 4 °C), and polymerized under UV for 3 days at 4 °C. Ultrathin sections (85 nm) were made with a ultracut (Leica EM UC7). After 1 h saturation with 0.1 M Tris, 150 mM NaCl in 1% BSA and 1% NGS1, immunolabeling was carried out using Polβ antibody (ab26343, Abcam). Polβ labeling was revealed with a goat anti-rabbit IgG gold conjugate (18 nm in diameter) (Jackson ImmunoResearch Laboratories). Staining of the ultrathin sections was carried out using 2% uranyl acetate in 50% ethanol. Labeling was observed under a Zeiss EM 900 electron microscope and acquisition realized with camera gatan (Orius SC 1000). Three sections per hippocampus, at the CA1 level, were observed with Zeiss EM 900 microscope and acquisition (100–200 images per section) realized with camera gatan (Orius SC 1000).

### Human brains

Human brain samples were obtained from the Lille Neurobank, which was given to the French Research Ministry by the Lille Regional Hospital (CHRU-Lille) on August 14, 2008 under the reference DC- 2000-642. The Lille Neurobank fulfills criteria from the French Law on biological resources, including informed consent, ethics review committee, and data protection (article L1243–4 du Code de la Santé publique, August 2007). Frontal cortex sections from human non-dement control (Ctr) and Braak VI Alzheimer brains (AD) (*n* = 3 for each category) were used for immunofluorescence analysis (Table 1).

**Table 1:** Braak stages, region, age, gender, and post mortem interval of the brains used in the study

	Braak stage	Tissue	Age	Sex	PMI^a^ (hours)
Ctr#1	0	Frontal Cortex	72	F	72
Ctr#2	0	Frontal Cortex	47	M	39
Ctr#3	0	Frontal Cortex	74	M	48
AD#1	VI	Frontal Cortex	73	F	5
AD#2	VI	Frontal Cortex	76	F	22
AD#3	VI	Frontal Cortex	78	M	17

^a^*PMI* post mortem interval

### Isolation of CA1 hippocampal region or whole hippocampus from mouse brain tissues

Brain areas where tau pathology is localized in the Tg mouse brain, i.e. the CA1 subfield of the hippocampus in 6-month-old mice, or the whole hippocampus in 12-month-old mice, were dissected, snap frozen in liquid nitrogen, and stored at − 80 °C. For each region, analyses were systematically compared between Tg and Wt mice to highlight the effect of tau pathology.

The CA1 subfield of the hippocampus from Tg and Wt littermates mice (*n* = 3 for each category) was isolated from whole brain hemispheres. Coronal sections (1 mm) from frozen mouse brain hemisphere were cut using an acrylic mouse brain matrice and matrice blades. For each frozen slice with hippocampus, the cortex was removed and the CA1 region was dissected on ice using a scalpel under a binocular loop (Leica MZ75) following anatomical landmarks (Figure S10).

### Protein extracts

Mouse brain samples were lysed for 30 min in ice-cold RIPA buffer containing protease inhibitors and phosphatase inhibitors cocktails (Roche). Debris was removed by centrifugation at 20,000 g at 4 °C for 10 min. The supernatant was used for immunoblotting.

For BER assays, the brain samples were suspended in buffer containing 20 mM HEPES, pH 7.5, 50 mM KCl, 2 mM EGTA and Complete protease inhibitor (Roche) and were homogenized. Lysates were centrifuged at 800 g for 10 min and suspended (2 mg/ml) in 20 mM HEPES (pH 7.0), 150 mM KCl, 2 mM EGTA, 1% (w/v) CHAPSO (Sigma), and protease inhibitor mixture and incubated at 4 °C for 1 h with end-over-end rotation. The lysates were centrifuged at 100,000 g for 1 h, and the supernatants were collected.

### Western blot

Whole cell extracts were separated in Tris-glycine SDS gels and transferred onto PVDF membrane. Each experiment was done 3–5 times. The images are shown as three technical replicates of one biological replicate. The primary antibodies used were: Actin (A5441, Sigma-Aldrich), Ac-SOD2 (Lys122, ab214675, Abcam), Caspase-3 (9962, Cell Signaling), DRP1 (8570, Cell Signaling), GAPDH (sc25778, Santa Cruz Biotechnology), LC3 (NB600–1384, Novus Biologicals), OGG1 (T1851, Epitomics), OPA1 (67,589, Cell Signaling), PGC-1α (NBP1–04676, Novus Biologicals), DRP1 (8570S, Cell Signaling), Phospho-DRP1 (Ser616, 3455, Cell Signaling), PINK1 (ab23707, Abcam), Polβ (ABE1408, Millipore), SIRT3 (2627, Cell Signaling), SOD2 (13,194, Cell Signaling), Tau (A002401–2, Agilent), TFAM (H00007019-B01P, Abnova), VDAC1 (ab14734, Abcam), and Vinculin (ab129002, To detect OXPHOS complex assembly, we used an assembly-dependent total OXPHOS rodent antibody cocktail (ab110413, Abcam). The antibodies in the cocktail are against a subunit that is labile when its complex is not assembled.

### MtDNA copy number

Total DNA (i.e. nuclear and mitochondrial) was extracted from mice brain using QIAamp DNA mini kit (Qiagen). The number of mtDNA molecules, or mtDNA copy number, was determined by quantitative-PCR using SYBR Green Supermix PCR (Bio-Rad), following the manufacturer’s protocol, and StepOnePlus Real-Time PCR system (Life Technologies). Nucleotide sequence of the primers is provided in Fig.12S.

### MtDNA integrity analysis

MtDNA integrity was analyzed using a PCR-based method as described previously [3, 68, 92] Briefly, PCR products were separated in agarose gel and the relative intensity of the amplicons, reflecting the level of integrity of the mtDNA templates, was measured using Image J. The nucleotide sequence of the PCR primers are shown in Fig. S12).

### AP site incision assay

Oligonucleotide containing a synthetic analogue of an AP-site 3-hydroxy-2- hydroxymethyltetrahydrofuran (shown as X) was annealed to its complementary oligonucleotide. The AP-site incision reaction was carried out in 20 mM HEPES-KOH pH 7.5, 50 mM KCl, 5 mM MgCl_2_, 1 mM DTT, 0.36 mg/ml BSA, 0.5% glycerol, using 1 μg extract and 2 pmol DNA substrate, at 30 °C for 5 min.

### Polβ activity assay (gap filling assay)

The assay for Polβ activity was carried out as follows. The extracts (5 μg) were incubated on ice for 5 min in 50 mM HEPES-KOH pH 7.5, 5 mM MgCl_2_, 70 mM KCl, 1 mM DTT, 50 μM of each dNTP, 0.36 mg/ml BSA, and 0.6 mM N-ethylmaleimide (NEM), before adding DNA substrate and initiation of DN synthesis. At the concentration used here, NEM inhibits non-Polβ DNA polymerases [4]. Polβ dependent DNA synthesis reaction was carried out at 37 °C for 10 min.

### 8-oxo-G excision assay

Reactions were carried out in 20 mM Tris-HCl pH 7.5, 0.1 M NaCl, 3 mM EDTA, 1 mM DTT, 0.1 mg/ml BSA, 0.5% glycerol, using 10 μg extract, 1 pmol DNA substrate, at 37 °C for 16 h.

### Uracil excision assay

Reactions were carried out in 20 mM Tris-HCl pH 7.5, 50 mM NaCl, 1 mM EDTA, 1 mM DTT, 0.1 mg/ml BSA, 5 μg extract, and 5 pmol DNA substrate at 37 °C for 15 min. Reactions were stopped by adding 1 M piperidine and heated at 90 °C for 20 min to cleave AP site generated after uracil removal.

The nucleotide sequences of all the oligonucleotides are shown in Fig. S12. DNA substrates were prepared by annealing the complementary oligos in 100 mM NaCl_2_, and 10 mM Tris-HCl, 90 °C for 5 min and slowly cooled to room temperature. All the reactions were terminated by adding loading buffer (10 mM EDTA, 95% formamide, 0.01% bromophenol blue, 0.01% xylene cyanol) and heated at 95 °C for 5 min. The unrepaired substrates (S), and the repair products (P) were separated by electrophoresis in a 15% polyacrylamide gel (19:1 acrylamide:bis), and were visualized in Typhoon 9410 (Amersham Biosciences), and quantified using Image J.

### Statistics

Two-tailed, unpaired *t*-test was used for statistical analysis of WB, transmission electron microscopy, immunofluorescence, BER activity and cell density (GraphPad Prism 7). The normality of data was tested by Q-Q plotting. For WB and BER activity, each lane represents one biological replicate (i.e. one sample from one mouse), and the number of biological replicates is indicated with green and red dots on the graph. For transmission electron microscopy and immunofluorescence, each biological replicate corresponds to one mouse. The number of biological replicates is indicated in the legends.

For cell density, each biological replicate corresponds to one mouse. The number of biological replicates is indicated with green and red dots on the graph. The experimenters were not blinded. Data are presented as mean ± SEM, *P < 0.05; **P < 0.01; ***P < 0.001.

## Results

### Hippocampal neurons in six-month-old Tg mice are enriched with oligomerized tau

**Fig. 1:**
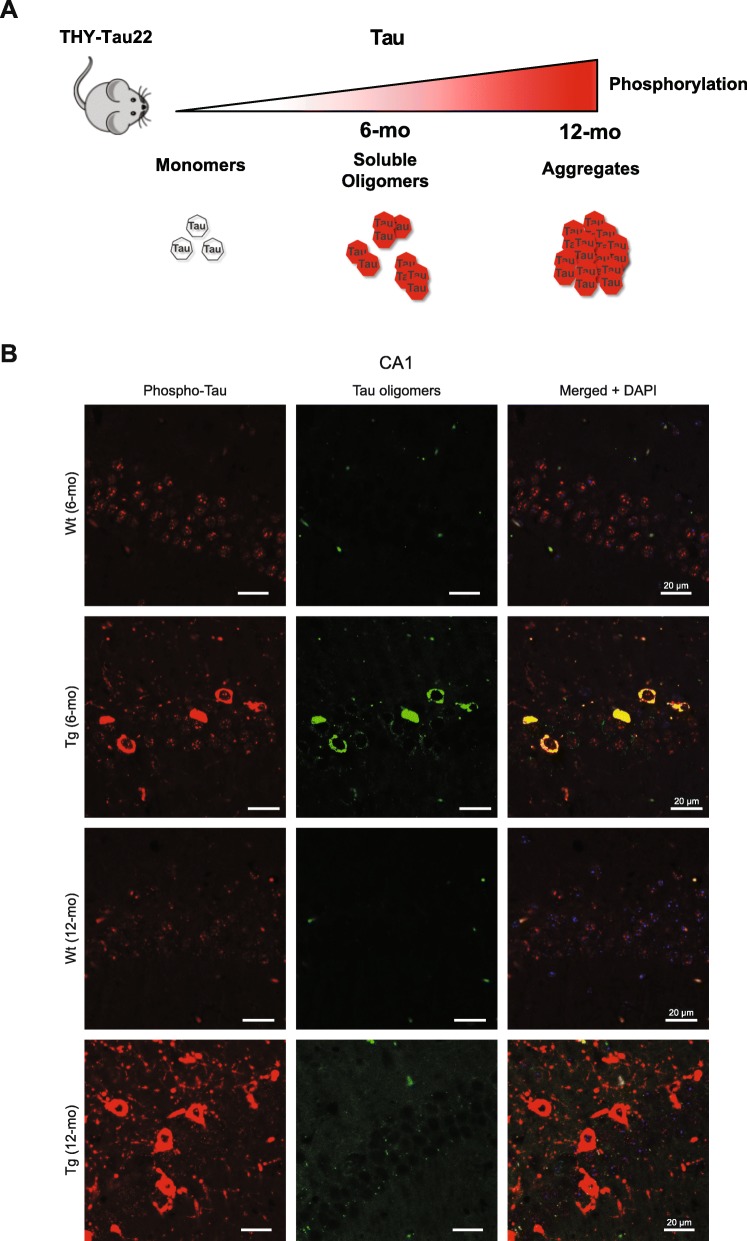
CA1 neurons in 6-mo Tg mice are enriched with tau oligomers. **a** Schematic of tau pathology progression in CA1 neurons of THY-Tau22 mice. **b** Representative images of sagittal CA1 sections from 6- and 12-mo Tg and Wt mice hippocampus (*n* = 3 for each mouse category). The sections were labeled with antibodies against pathological phospho-tau (pThr212), and tau oligomers (TOC1), respectively. The sections were analyzed by laser scanning confocal microscopy (z projection). Nuclei were visualized by DAPI staining. The scale bars represent 20 μm

The THY-Tau22 transgenic mouse model of AD-like tauopathy (Tg) develops progressive tau hyperphosphorylation and aggregation from 3 months of age that reaches a maximum at 12 months [70]. At 6 months of age, Tg mice accumulate soluble prefibrillar tau oligomers in the cytoplasm of CA1 neurons of the hippocampus (Fig. 1a) [6].

We monitored tau pathology by immunofluorescence in CA1 neurons in sagittal sections from Tg and Wt control mice using PT212 and TOC1 antibodies, which recognize phosphorylated Thr212 and soluble oligomerized tau, respectively [67, 86]. Hyperphosphorylated tau was detected in both six-month-old (6-mo) and 12-month-old (12-mo) Tg mouse CA1 neurons (Fig. 1b). Complete co-localization of cytoplasmic signals from PT212 and TOC1 antibodies was detected in CA1 neurons from 6-mo Tg mice (Fig. 1b). Contrary to PT212, TOC1 labeling was detected in almost all neurons in 6-mo Tg mice, but was no longer detectable in 12-mo Tg mice (Fig. 1b). Thus, prefibrillar, oligomeric tau is prevalent in Tg mice CA1 neurons early in the course of tau pathology but is not detectable at a later stage when tau pathology has reached its maximum [70].

### Altered mitochondrial morphology and the abundance of OXPHOS subunits in Tg mouse hippocampal neurons

**Fig. 2:**
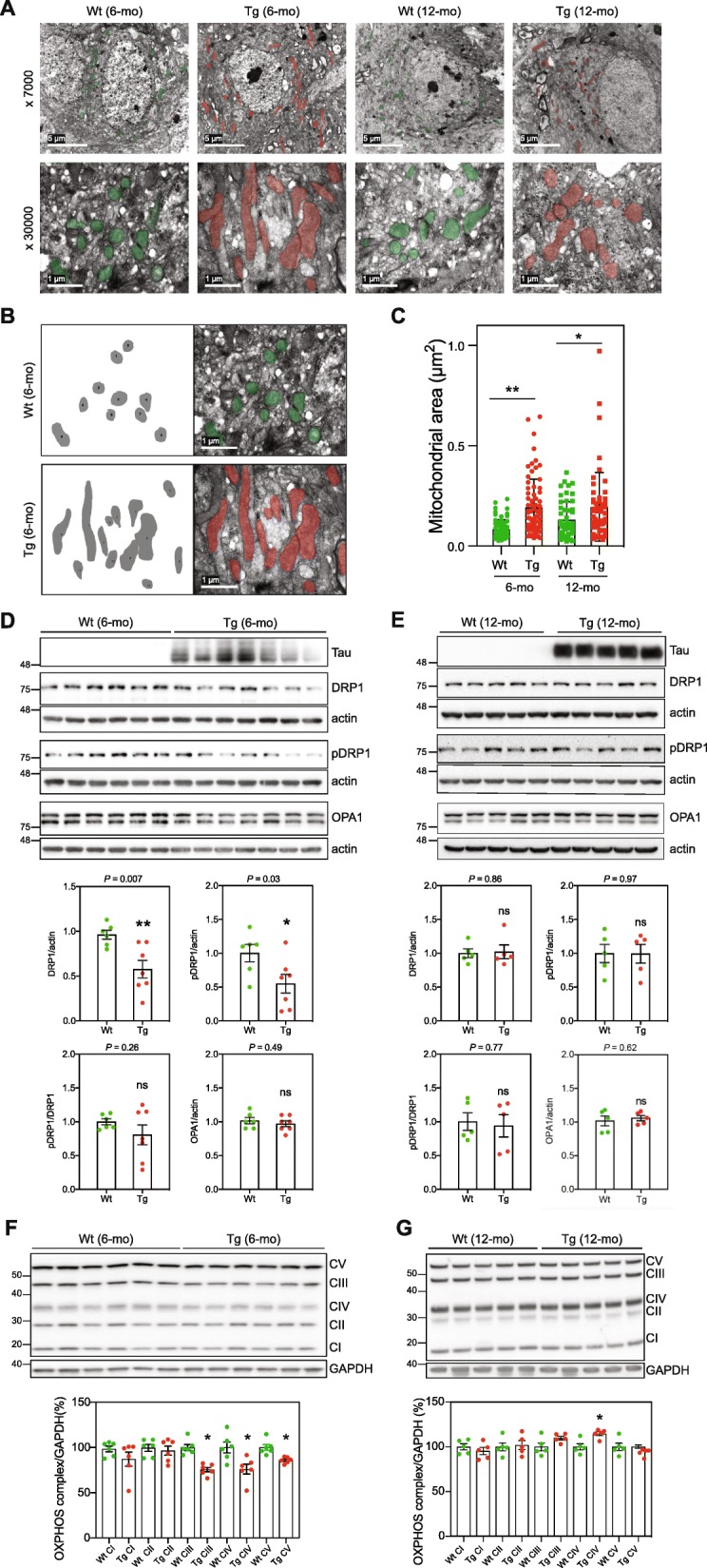
CA1 neurons in Tg mice show altered mitochondrial morphology. **a** Representative TEM images of CA1 sections from 6- and 12-mo Tg and Wt mice hippocampus (*n* = 3 for each mouse category). Mitochondria are highlighted by transparent colored overlay. The scale bars represent 5 μm or 1 μm. **b** Illustration of the quantification of mitochondrial area from TEM images. **c** Graph shows the mean of mitochondrial area per mouse category (mitochondria: 6-mo-Wt *n* = 63; 6-mo-Tg *n* = 51; 12-mo-Wt *n* = 72; and 12-mo Tg *n* = 54). Data is presented as mean ± SEM (*P = 0.039; **P = 0.0077). **d** WB analysis of extracts from 6-mo Wt and Tg mice CA1 (WT, *n* = 6; Tg, *n* = 7), using antibodies against mitochondrial fission and fusion proteins: DRP1, pDRP1 (S616) and OPA1. Actin was used as loading control. **e** WB analysis of extracts from 12-mo Wt and Tg mice hippocampi (Wt, *n* = 5; Tg, *n* = 5). Actin or Vinculin were used as loading control. Quantifications are shown in graphs. Data is presented as mean ± SEM (*P < 0.05; **P < 0.01). **f** WB analysis of extracts from 6-mo Wt and Tg mice CA1 (Wt, *n* = 6; Tg, *n* = 6) using an antibody cocktail against selected OXPHOS assembly subunits (CI: NDUFB8; CII: SDHB; CIII, MTCO1; CIV: UQCRC2; CV: ATP5A). **g** Graph showing the quantification results normalized to GAPDH. Data are shown as mean ± SEM (*P < 0.05)

Mitochondrial morphology is a key indicator of the state of cellular and tissue mitochondrial function and health [1, 71, 92, 93]. Transmission electron microscopy (TEM) analysis revealed an enrichment of elongated hippocampal mitochondria in Tg mice compared with Wt mice, which was more pronounced in 6-mo Tg mice than in 12-mo Tg mice (Fig. 2a, b and c).

Mitochondria undergo fusion and fission resulting in elongated or smaller mitochondria, respectively. Key proteins that regulate mitochondrial fission and fusion include the cytosolic dynamin-related protein 1 (DRP1) and the mitochondrial inner membrane protein optic atrophy 1 (OPA1), respectively [13]. The level of DRP1 in CA1 from 6-mo Tg mice was significantly lower than in the CA1 from 6-mo Wt mice (Fig. 2d). Phosphorylation of DRP1 at S616 (pDRP1) promotes the recruitment of DRP1 onto mitochondria and initiates the fission process [79]. There was no difference in the ratio of pDRP1/DRP1 suggesting that at 6 months of age, the reduced fission in Tg mice CA1 is caused by downregulation of DRP1 expression or increased protein turnover (Fig. 2d). Neither the amount of total DRP1, nor the level of pDRP1, were different between 12-mo Tg and Wt mouse hippocampi (Fig. 2e), suggesting that the reduced level of DRP1 in Tg mice was age dependent, occurs at early stages of tau pathology, and may contribute to the observed increased number of elongated mitochondria in Tg mouse hippocampi (Fig. 2a and b). The level of the mitochondrial inner membrane fusion protein OPA1, however, was not significantly different between Tg and Wt mice at any age, but a decrease was trending lower (*P* = 0.06) in 12-mo Tg mice (Fig. 2d and e).

The structure of the mitochondrial inner membrane determines the assembly and efficiency of the mitochondrial oxidative phosphorylation (OXPHOS) complexes (CI-CV) [17], linking mitochondrial morphology to mitochondrial function [93]. WB analysis showed reduced levels of CIII, CIV, and CV in 6-mo Tg hippocampus compared with the Wt mice (Fig. 2f). In 12-mo Tg mice, however, the level of CIV was higher compared with Wt mice while the levels of CIII and CV were not (Fig. 2g).

Thus, at 6 months of age, hippocampal neurons in Tg mice are accumulating tau oligomers, and displaying alterations in the structure and morphology of mitochondria. These observations, link tau oligomerization to altered mitochondrial fission and to OXPHOS structure and function.

### Elevated mitochondrial stress response in Tg mice hippocampus

**Fig. 3:**
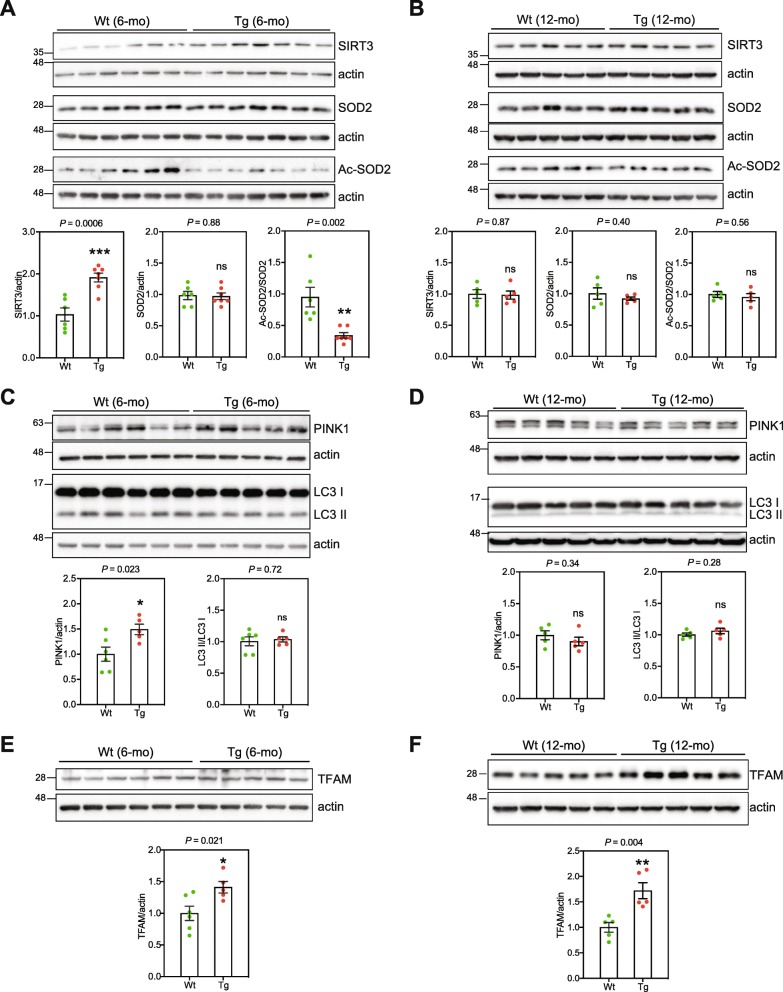
CA1 Tg mice undergo mitochondrial stress. **a** WB analysis of extracts from 6-mo Wt and Tg mice CA1 (Wt, *n* = 6; Tg, *n* = 7), using antibodies against SIRT3, SOD2 and acetylated SOD2. **b** WB analysis of extracts from 12-mo Wt and Tg mice hippocampi (Wt, *n* = 5; Tg, *n* = 5). Data are presented as mean ± SEM (**P < 0.01; ***P < 0.001). WB analysis of extracts from 6-mo Wt and Tg mice CA1 (Wt, *n* = 6; Tg, *n* = 5) (**c**), and from 12-mo Wt and Tg mice hippocampi (Wt, *n* = 5; Tg, *n* = 5) (**d**) for PINK1 and LC-3. Data are presented as mean ± SEM, *P < 0.05. WB analysis of extracts from 6-mo WT and Tg mice CA1 (WT, *n* = 5; Tg, *n* = 6) (**e**), and from 12-mo WT and Tg mice hippocampi (WT, *n* = 5; Tg, *n* = 5) (**f**) for TFAM. Data are presented as mean ± SEM (*P < 0.05), **P < 0.01). Actin was used as loading control

Mitochondrial dysfunction is frequently associated with mitochondrial stress phenotypes such as increased non-enzymatic acetylation of mitochondrial proteins typically resulting in altered function of the modified proteins [27, 87, 92]. Sirtuin 3 (SIRT3) is a mitochondrial NAD^+^-dependent deacetylase that maintains mitochondrial homeostasis under stress conditions by removing acetylation lesions from mitochondrial proteins [15, 55, 87]. WB analysis showed that the amount of SIRT3 in CA1 from 6-mo Tg mice was markedly higher than in CA1 from Wt mice (Fig. 3a), probably in response to perpetual mitochondrial stress.

Superoxide dismutase 2 (SOD2) is an important mitochondrial antioxidant enzyme. SIRT3 deacetylates and activates SOD2 [14, 80]. The level of acetylated SOD2, but not total SOD2, was significantly lower in 6-mo Tg mice CA1 compared with CA1 from Wt mice (Fig. 3a), consistent with higher levels of SIRT3. There were, however, no differences in the level of SIRT3, total SOD2 or acetylated SOD2 in 12-mo mice (Fig. 3b).

Mitophagy is the process of selective autophagic elimination of mitochondria. PTEN-induced putative kinase 1 (PINK1) accumulates on the outer mitochondrial membrane of damaged mitochondria with impaired membrane potential, thereby targeting the damaged mitochondria for mitophagy and lysosomal engulfment and degradation [36]. CA1 extracts from 6-mo but not from 12-mo Tg mice, contained higher amounts of PINK1 than Wt mice (Fig. 3c, d). This suggests the presence of more depolarized dysfunctional mitochondria in 6-mo Tg CA1 neurons.

Lipidated LC3 (LC3-II) facilitates substrate uptake by autophagosomes through binding to autophagy receptors. WB analysis of the ratio between nonlipidated LC3I/ to lapidated LC3-II has been used as a marker to monitor autophagic flux and response [31, 40]. There were no detectable differences in LC3-I/LC3-II ratio between Tg and Wt mice CA1 extracts, suggesting a similar basal autophagic flux (Fig. 3c, d).

The mitochondrial transcription factor A (TFAM) is an abundant protein with a central role in the initiation of mtDNA transcription and regulation of mtDNA replication [28]. Recently, cells from the long-lived Snell dwarf mice were shown to increase the expression of TFAM which improved mitochondrial stress response probably contributing to their increased lifespan [63]. The level of TFAM was higher in CA1 from Tg mice at both six and 12 months of age compared with Wt mice (Fig. 3e and f). WB analysis of whole CA1 extracts for VDAC1 showed no significant differences in mitochondrial mass in CA1 from 6-mo Tg and Wt mice (Suppl. Figure 1), indicating that TFAM was specifically increased in Tg mice CA1.

The transcriptional co-activator PGC-1α is an important regulator of mitochondrial biogenesis [69]. There were no detectable differences in the expression of PGC-1α between Tg and Wt mice (Suppl. Figure 2). Notably, the activity of PGC-1α is controlled by phosphorylation and acetylation [35, 61], detection of which often requires an immunoprecipitation step [26], which was not attempted here because of material limitation.

Thus, 6-mo Tg mice display altered expression and activity of SIRT3 with decreased ac-SOD2 and increased expression of PINK1, while TFAM expression was higher in Tg mice than the control mice at both ages. These results are all consistent with response to mitochondria experiencing elevated stress.

### The levels of the mitochondrial protein ANT1 and VDAC1 are markedly changed in Tg mouse CA1 neurons with tau lesion

**Fig. 4:**
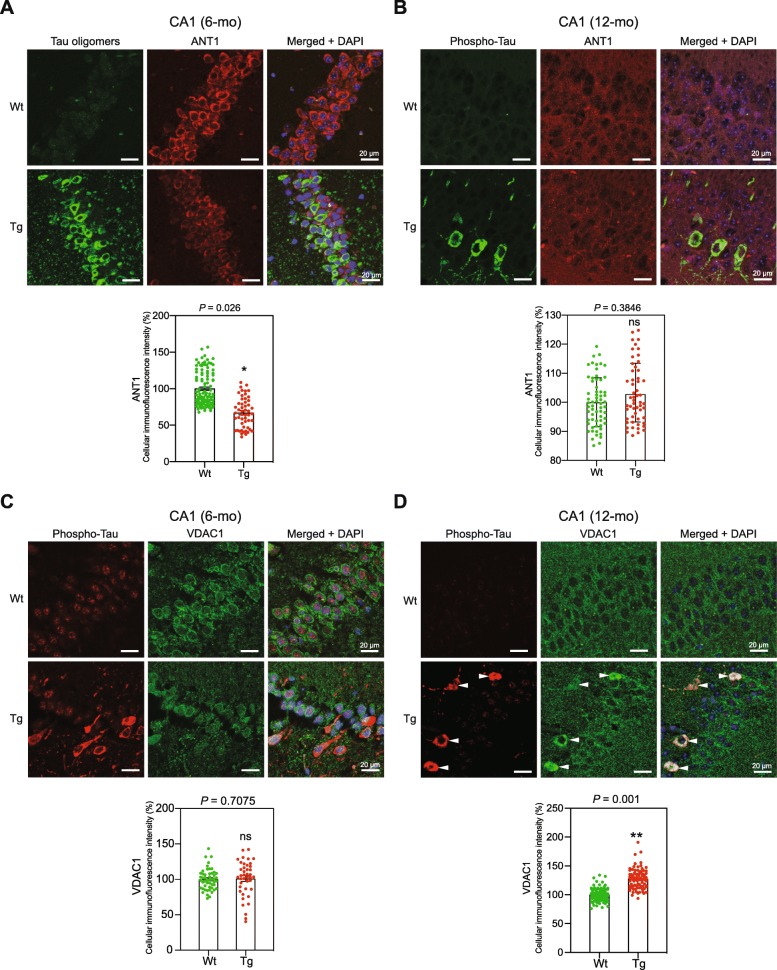
Altered levels of the mitochondrial proteins ANT1 and VDAC1 in Tg mice. Representative images of sagittal CA1 sections from 6- (**a**), and 12-mo (**b**), Tg and Wt mice hippocampi. The sections were labeled using TOC1 (against soluble tau oligomers) (**a**), pathological phosphorylated tau AT8 (against the phosphorylated tau at Ser202/Thr205) (**b**), and anti-ANT1 antibodies (*n* = 3 for each mouse category). Immunofluorescence signals were analyzed using laser scanning confocal microscopy (z projection). Nuclei were visualized with DAPI staining. The scale bars represent 20 μm. The cellular ANT1 fluorescence intensity was quantified within CA1 cells from 6- and 12-mo Wt and Tg hippocampi (cells: 6-mo Wt, *n* = 106; 6-mo Tg, *n* = 52; 12-mo Wt, *n* = 63; 12-mo Tg, *n* = 55). Graph shows the mean of cellular fluorescence per mouse category. Data are presented as mean ± SEM (*P < 0.05). Representative images of sagittal CA1 sections from 6- (**c**) and 12-mo (**d**) Wt and Tg mice hippocampi. The sections were labeled with antibodies against phosphorylated tau (pThr212), and VDAC1 (*n* = 3 for each mouse strain). Immunofluorescence signals were analyzed using laser scanning confocal microscopy (z projection). Nuclei were detected with DAPI staining. The scale bars represent 20 μm. The cellular VDAC1 fluorescence intensity was quantified within CA1 cells (6-mo Wt, *n* = 50; 6-mo Tg, *n* = 41; 12-mo Wt, *n* = 90; 12-mo Tg, *n* = 118). Graph shows the mean of cellular fluorescence per mouse category. Data are presented as mean ± SEM (**P < 0.01)

Adenine nucleotide translocator 1 (ANT1) is a highly abundant mitochondrial protein with important roles in the regulation of ADP/ATP exchange and mitochondrial membrane potential [8]. WB analysis did not show any differences in the ANT1 levels in lysates from 6- and 12-mo Tg and age-matched WT mice (data not shown). Immunofluorescence analysis, however, showed that the level of ANT1 was significantly reduced in 6-mo Tg mice CA1 neurons displaying high levels of tau oligomers compared with the Wt mice CA1 neurons (Fig. 4a). No differences were detected in 12-mo animals (Fig. 4b).

Voltage-dependent anion channel 1 (VDAC1), is an abundant mitochondrial outer membrane protein that plays central roles in various mitochondrial function [11]. The overall level of VDAC1 in 6-mo Tg mice CA1 was comparable to that in Wt mice (Fig. 4c, and Suppl. Figure 1). In 12-mo Tg mice CA1, however, the level of VDAC1 was significantly higher in neurons that also displayed high levels of cytoplasmic hyperphosphorylated tau compared with the rest of CA1 cells (Fig. 4d). Thus, the expression of mitochondrial proteins ANT1 and VDAC1 in Tg mice CA1 neurons are differently affected by age and the status of tauopathy.

### CA1 from 6-mo Tg mice contain increased copies of mtDNA molecules

The number of mtDNA molecules in a mitochondrion correlates with the cellular energy demand of the cell and ATP production and has been used to assess the state of mitochondrial function [16, 30]. CA1 from 6-mo (Suppl. Figure 3A) but not from 12-mo Tg mice (Suppl. Figure 3B) contained higher mtDNA copy numbers compared with CA1 from the Wt mice.

There were no significant differences in ATP levels in CA1 from 6-mo Tg mice compared to CA1 from Wt mice (Suppl. Figure 4), suggesting comparable levels of ATP production and perhaps consumption despite the morphological and mtDNA copy number differences.

### CA1 extracts from 6-mo Tg mice display elevated BER activity

**Fig. 5:**
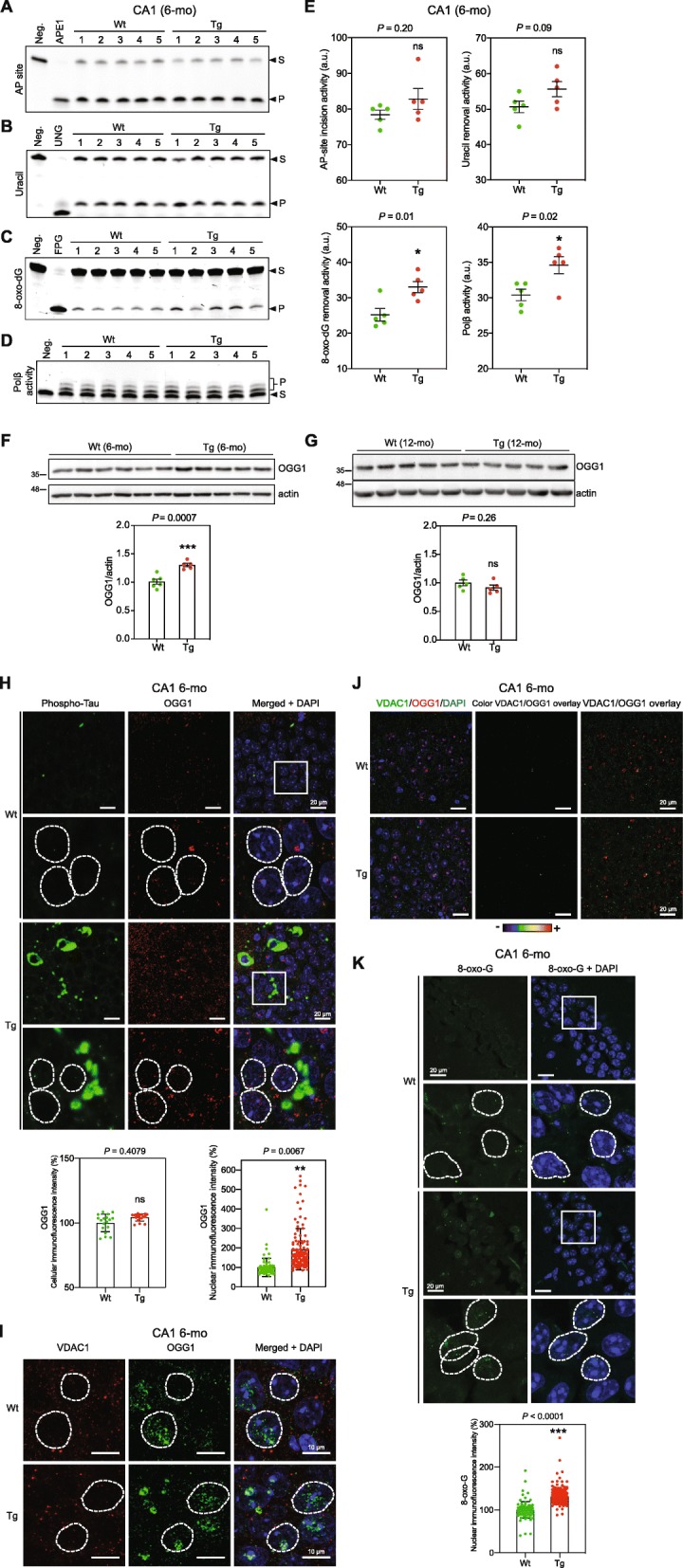
Increased levels of BER activity, 8-oxo-G base lesion, and OGG1 DNA glycosylase in CA1 neurons in 6-mo Tg mice. Biochemical analysis of BER activity in CA1 extracts from 6-mo Tg and Wt mice. **a** AP-site incision activity. Recombinant APE1 protein was used as a positive control. **b** Uracil removal activity. Purified recombinant UNG was used as a positive control. **c** 8-oxo-dG removal activity. Formamidopyrimidine DNA glycosylase (FPG) was used as a positive control. **d** Polβ nucleotide incorporation activity. **e** Quantifications of A-D**,** data are presented as mean ± SEM (*P < 0.05). WB analysis of CA1 extracts from 6-mo Wt (*n* = 5) and Tg (*n* = 6) mice (**f**), and hippocampus extracts from 12-mo Wt (*n* = 5) and Tg (*n* = 5) mice (**g**) for OGG1. Actin was used as a loading control. Quantification results are shown in the graph below. Data are presented as mean ± SEM (***P < 0.001). **h** Representative images of sagittal CA1 sections from 6-mo Wt and Tg mice. The sections were co-labeled with AT8 and anti-OGG1 antibodies (*n* = 3 for each mouse strain). The immunofluorescence signals were analyzed by laser scanning confocal microscopy (z projection). DAPI staining was used to visualize nuclei. Representative nuclei are delimitated by white dashed lines. The scale bars represent 20 μm. The cellular and nuclear OGG1 fluorescence intensity was quantified within CA1 cells (cells: Wt, *n* = 18; Tg, *n* = 18)(nuclei: Wt, *n* = 68; Tg, *n* = 116). Graph shows the mean of cellular or nuclear fluorescence per mouse category. Data are presented as mean ± SEM (**P < 0.01). **i** Possible mitochondrial localization of OGG1 was determined by labeling sagittal CA1 sections from 6-mo Wt and Tg mice with anti-VDAC1 and anti-OGG1 antibodies (*n* = 3 for each mouse strain). Immunofluorescence labeling was analyzed using laser scanning confocal microscopy (single confocal section)**.** DAPI staining was used to visualize nuclei. The scale bars represent 20 μm. Representative nuclei are delimitated by white dashed lines. The scale bars represent 10 μm. **j** Overlay of VDAC1 and OGG1 Immunofluorescence signals. The scale bars represent 20 μm. **k** Representative images of sagittal CA1 sections from 6-mo Wt and Tg mice hippocampi (*n* = 3 for each mouse strain). The sections were labeled with the 8-oxo-G antibody after RNAse pretreatment as described before [84] to selectively target oxidative DNA lesions (8-oxo-G). Immunofluorescence signals were analyzed using laser scanning confocal microscopy (z projection). Nuclei were detected with DAPI staining. Representative nuclei are delimitated by white dashed lines. The scale bars represent 20 μm. The intensity of the nuclear 8-oxo-G fluorescence signals was quantified within CA1 cells from 6-mo Wt and Tg hippocampi (nuclei: 6-mo Wt, *n* = 98; 6-mo Tg, *n* = 343). Graph shows the mean of cellular or nuclear fluorescence per mouse category. Data are presented as mean ± SEM (***P < 0.001). S, substrate; P, repair product

BER is the prominent pathway for repair of oxidative DNA damage and is active in both the nucleus and mitochondria [2, 78]. To test the biochemical effects of the observed mitochondrial stress in Tg mice, we carried out BER analyses in CA1 extracts. 8-oxo-G removal and DNA polymerase β (Polβ) repair DNA synthesis activities were markedly higher in CA1 extracts from 6-mo Tg mice compared with the Wt mice (Fig. 5c, d), but not in hippocampal extracts from 12-mo Tg mice (Suppl. Figure 5). These results suggest a tau-dependent and age-specific increase in BER activity in CA1, perhaps as a part of the cellular response to oxidative stress.

### Increased levels of nuclear OGG1 and 8-oxo-G in 6-mo Tg mice hippocampal neurons

8-oxo-guanine DNA glycosylase (OGG1) is the major enzyme for removal of 8-oxoG lesions from DNA. WB analysis showed a significantly higher amounts of OGG1 in hippocampal extracts from 6-mo but not from 12-mo Tg mice compared with Wt mice (Fig. 5f and g), corroborating the biochemical results (Fig. 5c, and Suppl. Figure 5).

Immunofluorescence analysis of CA1 sections showed significantly increased levels of OGG1 mainly in the nucleus of CA1 neurons in 6-mo Tg mice (Fig. 5h). Co-staining of OGG1 and VDAC1 did not show discernable co-localization signals, suggesting the absence or very low levels of OGG1 in mitochondria in CA1 neurons in 6-mo Tg mice (Fig. 5i and j).

CA1 hippocampal neurons from 6-mo Tg mice showed a higher level of 8-oxo-G base lesion signal compared with Wt control mice illustrating increased oxidative DNA damage (Fig. 5k). 8-oxo-G signal was restricted to nuclei suggesting that mtDNA did not contain discernible amounts of oxidative base lesions.

Thus, 6-mo Tg mice CA1 hippocampal neurons undergo elevated oxidative stress. The increased 8-oxo-G removal activity (Fig. 5c) and the levels of OGG1 protein (Fig. 5f) are likely part of the cellular response to increased oxidative stress.

### Altered level and intracellular distribution of Polβ in 6-mo Tg mouse hippocampal neurons and human AD brain

Polβ is a key BER protein, and likely the rate limiting one. It is predominantly a nuclear protein, but was recently detected in the mitochondria where it is likely involved in mtDNA repair [65, 76].

**Fig. 6:**
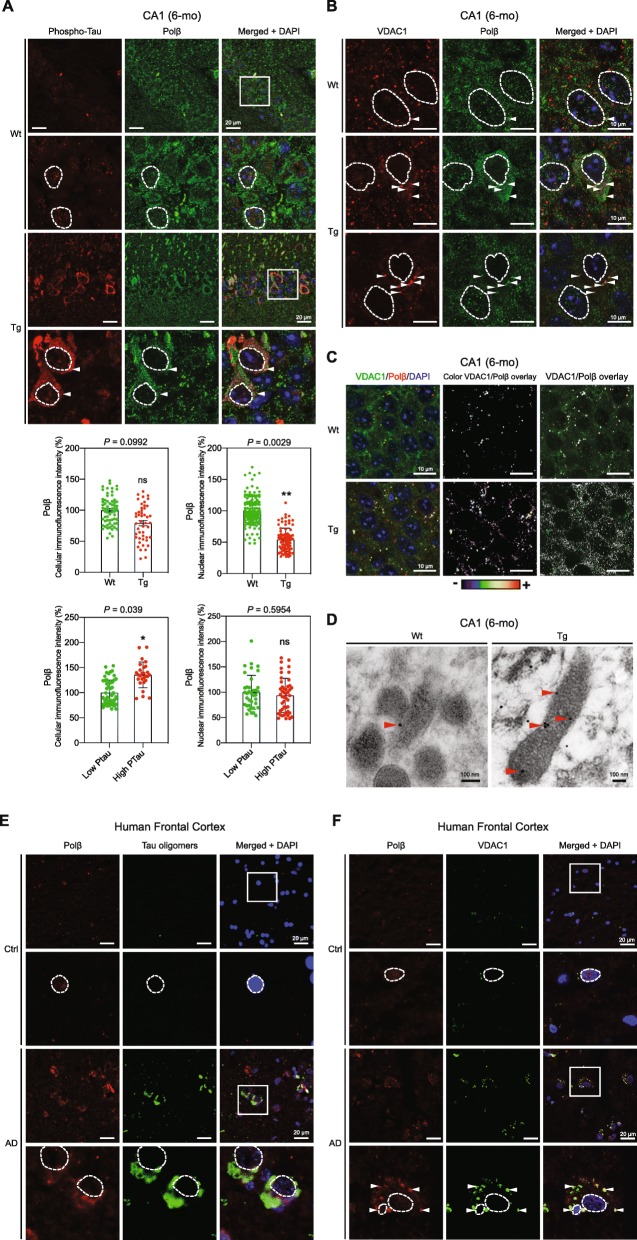
Cytoplasmic and mitochondrial accumulation of Polβ in 6-mo Tg mice hippocampal neurons. **a** Representative images of sagittal CA1 sections from 6-mo Wt and Tg mice hippocampi (*n* = 3 for each phenotype). The sections were co-labeled with anti-phospho-tau (AT8), and Polβ antibodies (*n* = 3 for each mouse category). Immunofluorescence signals were analyzed using laser scanning confocal microscopy (z projection). Nuclei were detected with DAPI staining. Representative nuclei are delimitated by white dashed lines. The scale bars represent 20 μm.. The intensity of the cellular and nuclear Polβ fluorescence signals was quantified within CA1 cells from 6-mo Wt and Tg hippocampi (cells: Wt, *n* = 64; Tg, *n* = 47; nuclei: Wt, *n* = 142; Tg, *n* = 88). Graph shows the mean of cellular or nuclear fluorescence per mouse category. Data are presented as mean ± SEM (*P < 0.05; **P < 0.01). The intensity of the cellular and nuclear Polβ fluorescence signals was separately quantified within CA1 cells from 6-mo Tg hippocampi with either high (High Ptau) or low phospho-tau (Low Ptau) levels (cells: Low Ptau *n* = 53, High PTau *n* = 27; nuclei Tg: Low Ptau *n* = 37, High PTau *n* = 47). Data are presented as mean ± SEM (*P < 0.05; **P < 0.01). The cut off value of fluorescence between high and low phospho-tau levels has been arbitrary chosen based on visual evaluation of the cytoplasmic phospho-tau labeling. **b** Mitochondrial localization of Polβ was assessed by labeling sagittal CA1 sections from 6-mo Wt and Tg mice with VDAC1 and Polβ antibodies (*n* = 3 for each mouse strain). Immunofluorescence signals were analyzed by laser scanning confocal microscopy (single confocal section). The scale bars represent 10 μm. Representative nuclei are delimitated by white dashed lines. White arrows point VDAC1 and Polβ co-localization. **c** Overlays of immunofluorescence labeling of VDAC1 and Polβ. **d** Representative immunoelectron microscopy images of CA1 sections from 6-mo Tg and Wt mice hippocampus. The sections were labeled with Polβ antibodies (*n* = 3 for each mouse strain). The scale bars represent 100 nm. Red arrows point Polβ localization. **e** Representative images of frontal cortex sections from human control (Ctr) and Braak VI Alzheimer frontal cortex (AD). The sections were labeled with the tau oligomer antibody, TOC1, and anti-Polβ antibody (*n* = 3 for each category). Immunofluorescence signals were analyzed by laser scanning confocal microscopy (z projection). Nuclei were detected with DAPI staining. Representative nuclei are delimitated by white dashed lines. The scale bars represent 20 μm. **f** Immunofluorescence labeling of the same sections with antibodies against Polβ and VDAC1 demonstrates mitochondrial localization of Polβ. Immunofluorescence signals were analyzed by laser scanning confocal microscopy (single confocal section). Nuclei were detected with DAPI staining. Representative nuclei are delimitated by white dashed lines. The scale bars represent 20 μm. White arrows point VDAC1 and Polβ localization

Because Polβ activity was increased in CA1 hippocampus from 6-mo Tg mice (Fig. 5d), we examined the effect of early tau pathology on the expression and cellular localization of Polβ within CA1 neurons. WB analysis and immunofluorescence analysis of CA1 sections showed no significant differences in Polβ levels in, respectively, CA1 extracts and CA1 cells from Tg and WT mice (Suppl. Figure 6, Fig. 6a). However, the level of nuclear Polβ was significantly reduced in 6-mo Tg mice (Fig. 6a). This was not observed in 12-mo Tg mice (Suppl. Figure 7).

Moreover, an increased overall cellular Polβ fluorescence signal was observed in CA1 cells from 6-mo Tg mice with tau hyperphosphorylation (High Ptau) (Fig. 6a). Because the level of nuclear fluorescence of Polβ did not significantly change in these cells, this shows that the increased tau phosphorylation correlates with an increased level of cytoplasmic Polβ (Fig. 6a). While the overall level of Polβ is unchanged in Tg compared to Wt mice (Suppl. Figure 6, Fig. 6a), interestingly, Polβ translocates from the nucleus to the cytoplasm in CA1 cells with tau hyperphosphorylation in 6-mo Tg mice.

Immunofluorescence co-labeling of Polβ and VDAC1 revealed co-localization of cytosolic Polβ with VDAC1, suggesting that Polβ is located within or onto mitochondria in CA1 neurons in 6-mo Tg mice (Fig. 6b and c). Immunoelectron microscopy using anti-Polβ antibody showed increased Polβ signals within mitochondria in 6-mo Tg mice compared to Wt (Fig. 6d, and Suppl. Fig. 8), further demonstrating the accumulation of Polβ within mitochondria in CA1 cells with hyperphosphorylated/oligomerized tau in 6-mo Tg mice.

These results suggest a potential role of Polβ in mtDNA repair in CA1 cell. We tested this using a PCR-based method [68, 92]. This method, as used here, mostly detects AP-sites in DNA. There were no detectable differences in the level of mtDNA integrity in CA1 from 6-mo and 12-mo Tg and Wt mice (Suppl. Figure 9). This suggests that the enrichment of Polβ in mitochondria in the neurons with hyperphosphorylated/oligomerized tau may contribute to the preservation of the mtDNA integrity in those cells that is comparable to Wt mice CA1; however, this needs further investigation.

We next investigated the relevance of these findings to human tau pathology. We found specific cytoplasmic accumulation of Polβ in neurons positive for soluble tau oligomers in frontal cortex from human AD brains (Fig. 6e and Suppl. Fig. S11). Cytoplasmic Polβ labeling was detected in all the cells that also showed the accumulation of tau oligomers. Co-labeling of Polβ and VDAC1 in the same brain samples highlighted strong co-localization signal (Fig. 6f and Suppl. Fig. S11). Thus, our results demonstrate a correlation between extra nuclear and mitochondrial localization of Polβ and the presence of soluble oligomerized tau protein in Tg mouse CA1 (Fig. 6a, b, c and d) and in frontal cortex from human AD brains (Fig. 6e, f and Suppl. Fig. 11).

### Tau oligomerization and mitochondrial stress do not lead to significant CA1 neuronal loss

**Fig. 7:**
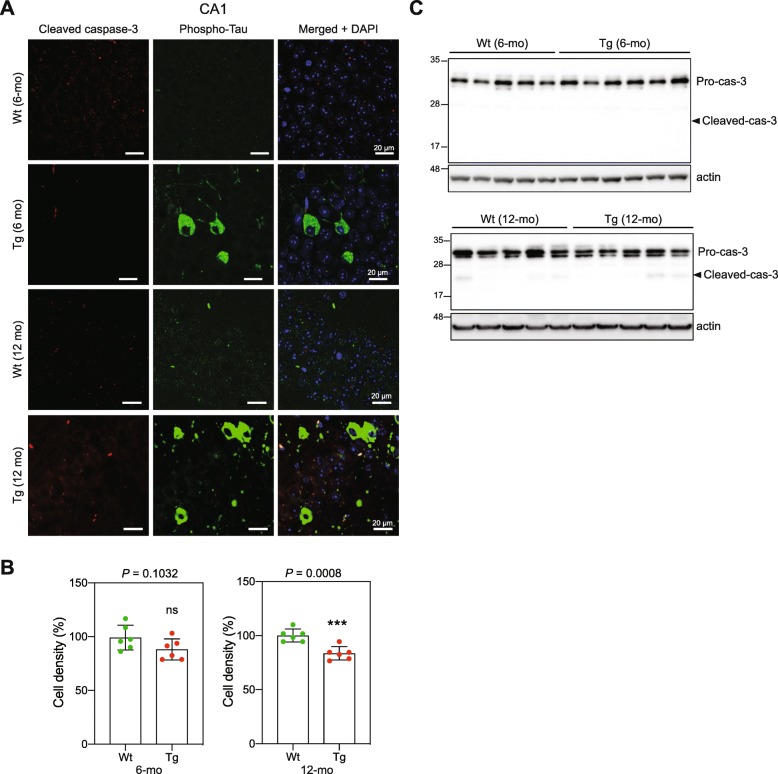
Accumulation of oligomeric tau in 6-mo Tg mice does not lead to detectable level of apoptotic cell death. Representative images of sagittal CA1 sections from 6- and 12-mo Wt and Tg mice hippocampi (*n* = 3 for each category), labeled with antibodies against the phospho-tau (AT8), and activated (cleaved) caspase-3 antibodies. Immunofluorescent signals were analyzed by LSC laser scanning confocal microscopy (z projection). Nuclei were visualized with DAPI staining. The scale bars represent 20 μm. **b** Quantification of nuclei (DAPI staining) per surface unit within CA1 sections from 6- and 12-mo Wt and Tg mice hippocampi (*n* = 10 for each mouse category). DAPI signals were analyzed by LSC laser scanning confocal microscopy (z projection). Graph shows the mean of nuclear fluorescence per mouse. Data are presented as mean ± SEM (***P < 0.001). **c** WB analysis of CA1 lysates from 6- and 12-mo mice and hippocampi for pro-caspase-3 and activate cleaved caspase-3. Actin was used as loading control

Mitochondrial dysfunction may trigger cell death by apoptosis [83]. Caspases are central mediators of neuronal apoptosis. Activated caspase-3 has been detected in hippocampus in AD mouse models, and was related to increased DNA damage and neuronal death [77], but also to early synaptic dysfunction without cell death [20]. Immunofluorescence analysis of CA1 sections did not show detectable levels of activated caspase-3 in Tg or in Wt mice (Fig. 7a). Rare caspase-3 activated apoptotic events may be difficult to detect. Thus, we quantified the DAPI-stained nuclei to determine the cell density in the CA1 section as a measure of cell loss. A slight but statistically significant decrease in cell density was detected in 12-mo Tg mouse CA1 compared with CA1 from 12-mo Wt mice (Fig. 7b). WB analysis showed very faint bands corresponding to the expected size of cleaved caspase-3 in CA1 extracts from 12-mo Wt and Tg mice (Fig. 7c). Together, these results suggest that tau oligomerization and the mitochondrial alterations observed in 6-mo Tg CA1 cells do not lead to a detectable level of CA1 neuronal loss; however, at later stages of tau pathology, some neuronal loss in CA1 may take place as previously described [70]. It is noteworthy, however, that the use of a stereological tool like optimized optical fractionator would be necessary for unbiased estimation of neuronal number [33].

## Discussion

**Fig. 8:**
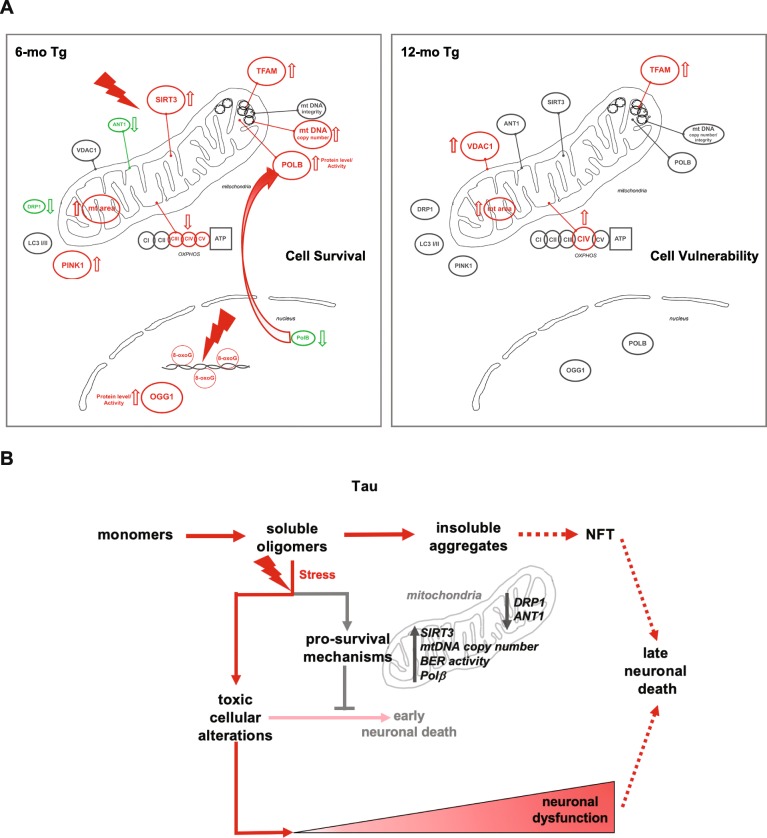
**a** Schematic summary of mitochondrial and BER alterations in hippocampus neurons from 6- and 12-mo Tg mice detected in this study. **b** Schematic roles of soluble tau oligomers in disease progression

In this study, we show that at early stages of tau pathology when soluble tau oligomers are prominent, hippocampal neurons undergo significant changes in mitochondrial homeostasis, DNA repair activity, and cytosolic and mitochondrial translocation of Polβ (Fig. 8a). These events likely promote cell survival and counter cellular stress caused by tau oligomers.

Prefibrillar oligomers have emerged as the more deleterious forms of tau rather than the previously thought larger tau aggregates [29, 32, 46, 60, 64, 84] and other pathogenic tau species in AD [46]. In cell models, oligomeric tau intermediates have been described as neuronal death inducers [29, 44]. Injection of recombinant human tau oligomers into mouse hippocampus produced mitochondrial respiratory chain dysfunction, activated the pro-apoptotic protein caspase-9 and induced neurodegeneration in the CA1 region [45]. However, if endogenous soluble tau oligomers induced cell death in AD brains, neurons with insoluble aggregates and NFT should not be observed. This suggests that in human brains, neurons with tau pathology develop efficient mechanisms to counter the cell toxicity caused by soluble tau oligomers and promote neuronal survival.

Consistent with this hypothesis, our results show that CA1 neurons in 6-mo Tg mice with prominent endogenous prefibrillar tau oligomers in the cytoplasm, activate a cellular stress response and show no evidence for increased cell death.

A recent study showed that the method of tau oligomers preparation and other experimental conditions might account for the observed cytotoxicity of tau oligomers [38]. Under improved experimental conditions, tau oligomers caused synaptotoxicity, increased ROS production, and impaired calcium homeostasis, but had no overt effects on cell viability [38]. These findings are largely in line with our conclusions. We cannot, however, rule out that some populations of neurons may fail to develop effective pro-survival mechanisms against tau oligomers-induced toxicity and die, thus, contributing to early neuronal loss observed in AD.

SIRT3 is the primary mitochondrial NAD^+^-dependent deacetylase [52]. In stressful conditions such as excitotoxicity and high ROS production, SIRT3 maintains mitochondrial homeostasis and promotes neuronal stress resistance and survival [21, 39, 66]. Oxidative and metabolic stress modulate the expression of SIRT3 [55]. A decrease in SIRT3 protein level was linked to mitochondrial dysfunction in AD brain [48]. Increased level and activity of SIRT3 (Fig. 3a) concomitant with the enrichment of prefibrillar tau oligomers in 6-mo Tg mouse CA1 region, suggests that CA1 neurons are undergoing stress and activate SIRT3 as part of a stress response to sustain mitochondrial function and to promote the survival of neurons developing tau pathology.

Mitochondria are structurally dynamic organelles and undergo fusion and fission resulting in elongated and smaller mitochondria, respectively. Fusion and fission are implicated in the elimination of abnormal mitochondria by mitophagy [81], maintaining mtDNA integrity [24], regulating mitochondrial morphology [51], and mitochondria distribution within the cell [50]. The central fission protein is the cytosolic DRP1. In 6-mo Tg mice hippocampal cells enriched with tau oligomers, the level of DRP1 was significantly reduced together with mitochondrial elongation linking altered expression of DRP1 to mitochondrial morphology changes early during tau pathology.

DRP1 interacts with hyperphosphorylated tau in AD brain [53]. Interestingly, reduced DRP1 and increased mitochondrial fusion protected against various tau-induced mitochondrial dysfunction in the cortex and hippocampus of a double mutant P301L tau and DRP1 heterozygous knockout mice [37]. Thus, the reduced DRP1 level and mitochondrial elongation may constitute a key mechanism to maintain mitochondrial homeostasis and to promote cell survival in 6-mo Tg mice CA1 hippocampal neurons. In 12-mo Tg mice, mitochondrial elongation decreased but persists despite DRP1 returning to Wt levels.

ANT1 transports ADP and ATP across the mitochondrial inner membrane. ANT1-deficient mice do not show overt brain pathology, and ANT1-deficient neurons are resistant to excitotoxicity induced death [49]. Thus, it is possible that the significant reduction in the expression of ANT1 in 6-mo Tg mice CA1 neurons (Fig. 4a) provides a neuroprotective function, maybe as part of a mitochondrial and neuronal stress response to tau oligomers.

The mitochondrial outer membrane protein VDAC1 plays central roles in regulating mitochondrial function and mitochondrial-related apoptosis [11]. VDAC1 has been linked to AD pathogenesis [73] and VDAC1 interaction with hyperphosphorylated tau negatively affected mitochondrial function [54]. The increased concentration of VDAC1 in hyperphoshorylated tau-expressing CA1 neurons in 12-mo Tg mice, but not in 6-mo Tg mice, is in line with a previous report showing high expression of VDAC1 in AD brains at advanced stages of the disease and in AD transgenic mouse brain [18]. Overexpression of VDAC1 induces apoptotic cell death in various cell types [90], consistent with the reduced cell density seen in 12-mo Tg CA1 neurons (Fig. 7b).

Thus, our results suggest that most of the neuro-protective stress responses in 6-mo Tg mice CA1 neurons are transient and effective in the early stages of tau pathology when tau oligomers are abundant. Notably, an adaptive pro-survival response triggered by mitochondrial stress has been recently described in human skeletal muscle cells [56].

8-oxo-G is the major oxidative DNA base lesion and is frequently used as a biomarker of the state of cellular stress and ROS generated DNA damage [22]. OGG1 is the major DNA glycosylase for the removal of 8-oxo-G from DNA [43]. Our results showed increased 8-oxo-G levels together with enhanced OGG1 protein level and 8-oxo-G repair activity in the 6-mo Tg mice CA1 neurons. Thus, we have identified a combination of increased DNA lesions and the activity that repairs those lesions as a novel consequence of tau oligomerization.

Polβ deficiency exacerbates AD phenotypes and mitochondrial dysfunction [34, 58, 77], and post- mortem brain extracts from AD patients showed impaired Polβ activity [88]. Polβ is the central nuclear BER DNA polymerase in mammalian cells. Mice with targeted disruption of Polβ die shortly after birth [74]. Histological examination of the embryos revealed a key role for Polβ in neurogenesis and neuronal development [74]. Polβ is predominantly present in the nucleus; however, recently, Polβ has also been detected in mitochondria where it contributes to mtDNA integrity [65, 76]. The localization of Polβ in mitochondria appears to be tissue dependent and controlled by an unknown mechanism, because it was found in mouse brain mitochondria but not in the liver mitochondria [76]. Thus, under normal physiological conditions, Polβ is present in the brain nuclei and mitochondria. However, the translocation of Polβ from the nucleus to mitochondria appears more prominent under cellular stress conditions generated by tau oligomerization (Fig. 6). These results suggest a protective function of mitochondrial localization of Polβ in the brain that may involve mtDNA repair [76]. However, other hitherto unknown functions cannot be ruled out and this is an interesting and important subject for investigation. Importantly, this is the first demonstration of mitochondrial localization of Polβ in the etiology of tauopathy and AD.

Tau oligomers are thought to be neurotoxic and cause mitochondrial dysfunction [44, 45]. Our results, however, reveal several protective mechanisms, e.g. increased SIRT3 (Fig. 3a), mtDNA copy number (Suppl. Figure 3), BER activity (Fig. 5c and d), and reduced DRP1 and ANT1 levels (Fig. 2d and 4a), in response to tau lesion, probably allowing neurons to prevent irreversible mitochondrial dysfunction and cell death (Fig. 8b). The benefit of these protective mechanisms early in tau pathology might, however, contribute to the long-term survival of neurons expressing tau pathology [59], and in that way contribute to the progression of AD and related tauopathies.

An important finding in our work is that almost all mitochondrial, as well as DNA damage and repair, phenotypes observed in 6-mo Tg mice hippocampus were reversed or corrected to the level of Wt mice when the mice were 12 months old. The protective response correlated to the presence of tau oligomers was not a secondary effect of tau overexpression, because the level of tau actually substantially increased over time (Fig. 2d, e).

In human AD brains, intraneuronal accumulation of soluble tau oligomers correlates with synaptic alterations, neuroinflammation and memory deficits [32, 42, 62]. Notably, synaptic loss, neuroinflammation, and memory deficit, but no cell loss, have been reported in 6 months old THY-Tau22 mice bred in the same lab (66, 44). This suggests that the endogenous soluble tau oligomers have a deleterious effect on neuronal function, unrelated to cell death. Altogether, our results suggest that in 6-mo THY-Tau22 mice, CA1 neurons develop mechanisms to protect against cell toxicity and cell death caused by soluble tau oligomers, but fail to prevent fully soluble tau oligomers from triggering cellular dysfunctions. Cellular alterations elicited by soluble tau oligomers will persist and participate in progressive neuronal dysfunction and behavioral deficits despite the decrease or loss of toxic oligomeric tau at later stages of the pathology (Fig. 8b).

A number of recent studies have investigated various intervention strategies to improve or correct key disease phenotypes in animal models of human neurodegenerative diseases including AD [25, 26, 34]. These studies have demonstrated mitochondria and DNA damage responses as potentially safe therapeutic targets. It will be interesting to investigate the effect of these drugs on the progression of tau pathology and neuronal stress response in THY-tau22 mouse.

In summary, our data suggests that early during the development of tau pathology, when soluble prefibrillar tau oligomers are prominent and cells undergo stress, CA1 hippocampal neurons activate pro-survival mitochondrial and DNA repair stress responses. However, these protective mechanisms may also allow the progressive accumulation of tau aggregates in affected neurons, and increasing risk of propagation of pathological forms of tau to neighboring neurons over time, as observed in AD. Notably, while extranuclear Polβ in hyperphosphorylated and oligomerized tau overexpressing cells may have a positive effect on mtDNA integrity, it may have a deleterious effect on nuclear BER whereby promoting genomic instability observed in early AD [7, 72, 84]. Thus, although soluble tau oligomers represent plausible targets for treatment [12], controlling pro-survival mechanisms as a therapeutic strategy needs careful investigation.

To our knowledge, this work provides the first description of multiple stress responses implicating mitochondrial homeostasis and BER early during the progression of tau pathology and tau oligomerization (Fig. 8). In our view, these results represent an important advance in the etiopathogenesis of tauopathies. We speculate that the activation of pro-survival stress response trigged by tau oligomers contributes to the progressive neuronal dysfunction and the strong age-associated clinical manifestations of tauopathies (Fig. 8a, b). Elucidating the underlying molecular mechanisms that control these events and the consequences of their failure to the progression of tau pathology and neuronal dysfunction warrants investigation.

## Supplementary information

**Additional file 1 **: **Figure S1.** Tau pathology does not affect mitochondrial mass in CA1 neurons. WB analysis of extracts from 6-mo Wt and Tg mice CA1 (Wt, *n* = 6; Tg, *n* = 5) (**A)**, and from 12-mo Wt and Tg mice hippocampi (Wt, *n* = 5; Tg, *n* = 6) **(B)** for VDAC1. Actin was used as loading control. Data are presented as mean ± SEM.

**Additional file 2 **: **Figure S2.** Tau pathology does not affect the expression of the key regulator of mitochondrial biogenesis Pgc1α**.** WB analysis of extracts from 6-mo Wt and Tg mice CA1 (Wt, *n* = 6; Tg, *n* = 5) (**A)**, and from 12-mo Wt and Tg mice hippocampi (Wt, *n* = 5; Tg, *n* = 6) **(B)** for PGC-1α. Actin was used as loading control. Data are presented as mean ± SEM.

**Additional file 3 **: **Figure S3.** Early tau pathology correlates with increased number of mtDNA molecules. **(A)** Quantitative-PCR analysis of mtDNA copy number was carried out in total DNA isolated from CA1 region from 6-mo Wt (*n* = 5) and Tg (*n* = 6) mice. *Nd1* and *16 s rRNA* are mitochondrial genes and *Hk2* is a nuclear gene. **(B)** Analysis of mtDNA copy number in total DNA from 12-mo Wt (*n* = 5) and Tg (*n* = 5) mice hippocampi. Data are presented as mean ± SEM (*P < 0.05).

**Additional file 4 **: **Figure S4.** ATP measurements in 6-mo Tg and Wt CA1. ATP was measured using a luciferase-based assay (ATPlite Luminescence Assay Kit, PerkinElmer), following the Manufacturer’s protocol. The content of ATP was normalized to protein content and presented as percentage of control.

**Additional file 5 **: **Figure S5.** BER activity is not changed in 12-mo Tg mice. Biochemical analysis of BER activity in hippocampal extracts from 12-mo Tg and Wt mice. **(A)** AP-site incision activity. Recombinant APE1 protein was used as a positive control. **(B)** Uracil removal activity. Purified recombinant UNG was used as a positive control. **(C)** 8-oxo-G removal activity Formamidopyrimidine DNA glycosylase (FPG) was used as a positive control. **(D)** Polβ nucleotide incorporation activity. **(E)** Quantifications of A-D**,** data are presented as mean ± SEM.

**Additional file 6 **: **Figure S6.** Polβ protein level is not changed in Tg mice. WB analysis of extracts from 6-mo Wt and Tg mice CA1 (Wt, *n* = 7; Tg, *n* = 6) (**A)**, and from 12-mo Wt and Tg mice hippocampi (Wt, *n* = 5; Tg, *n* = 5) **(B)** for Polβ. Actin was used as loading control. Data are presented as mean ± SEM.

**Additional file 7 **: **Figure S7.** Absence of cytoplasmic accumulation of Polβ in 12-mo Tg mice hippocampal neurons. Representative images of sagittal CA1 sections from 12-mo Wt and Tg mice hippocampi. The sections were co-labeled with anti-phospho-tau (AT8), and Polβ antibodies (*n* = 3 for each mouse category). Immunofluorescence signals were analyzed using laser scanning confocal microscopy (z projection). Nuclei were detected with DAPI staining. Representative nuclei are delimitated by white dashed lines. The scale bars represent 20 μm. The intensity of the cellular and nuclear Polβ fluorescence signals was quantified within CA1 cells from 12-mo Wt and Tg hippocampi (nuclei: Wt, *n* = 84; Tg, *n* = 66; cellular: Wt, *n* = 97; Tg, *n* = 98). Graph shows the mean of nuclear fluorescence per mouse category. Data are presented as mean ± SEM.

**Additional file 8 **: **Figure S8.** Increased mitochondrial Polβ in Tg mice hippocampal neurons. Representative immunoelectron microscopy images of CA1 sections from 6-mo Tg and Wt mice hippocampus. The sections were labeled with Polβ antibodies (*n* = 3 for each mouse category). The scale bars represent 100 nm. Red arrows point Polβ localization

**Additional file 9 **: **Figure S9.** PCR-based mtDNA damage analysis. **(A)** MtDNA damage analysis from 6-mo Wt (*n* = 5) and Tg (*n* = 6) mice CA1 region by long range PCR. **(B)** MtDNA analysis of 12-mo Wt (*n* = 5) and Tg (*n* = 5) mice hippocampi. Data are presented as mean ± SEM. Statistics were performed with unpaired two-tailed Mann-Whitney test (**P < 0.01)

**Additional file 10 **: **Figure S10.** Schematic representation of a coronal mouse hippocampal section. The dashed red line shows the dissected CA1 region.

**Additional file 11 **: **Figure S11.** Increased cytoplasmic and mitochondrial accumulation of PolB in neurons from AD brain. Representative images of frontal cortex sections from human control (Ctr) and Braak VI Alzheimer (AD) frontal cortex. Immunofluorescence signals were analyzed by laser scanning confocal microscopy. Nuclei were detected with DAPI staining. The scale bars represent 20 μm. **(A)** The sections were labeled with the tau oligomer antibody, TOC1, and anti-Polβ antibody (*n* = 3 for each category) (z projection) or **(B)** with antibodies against Polβ and VDAC1 (single confocale section).

**Additional file 12 **: **Figure S12.** List of oligonucleotides used in mtDNA analysis and in BER assays.
