# Supplementary figures and images for: Hippocampal tau oligomerization early in tau pathology coincides with a transient alteration of mitochondrial homeostasis and DNA repair in a mouse model of tauopathy
Source: Acta Neuropathol Commun. 2020 Mar 4;8:25. doi: 10.1186/s40478-020-00896-8 (PMC7057491; doi:10.1186/s40478-020-00896-8)

Figure S1

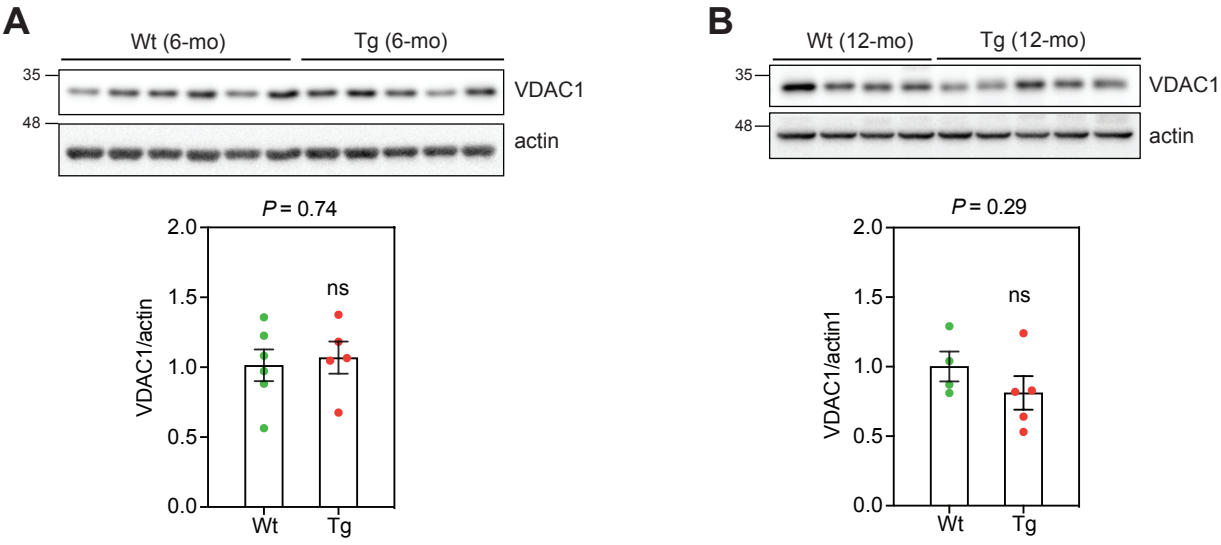

Supplement: Supplementary file 1 — Additional file 1 : Figure S1. Tau pathology does not affect mitochondrial mass in CA1 neurons. WB analysis of extracts from 6-mo Wt and Tg mice CA1 (Wt, n = 6; Tg, n = 5) (A), and from 12-mo Wt and Tg mice hippocampi (Wt, n = 5; Tg, n = 6) (B) for VDAC1. Actin was used as loading control. Data are presented as mean ± SEM. [file 40478_2020_896_MOESM1_ESM.pdf]

Figure S2

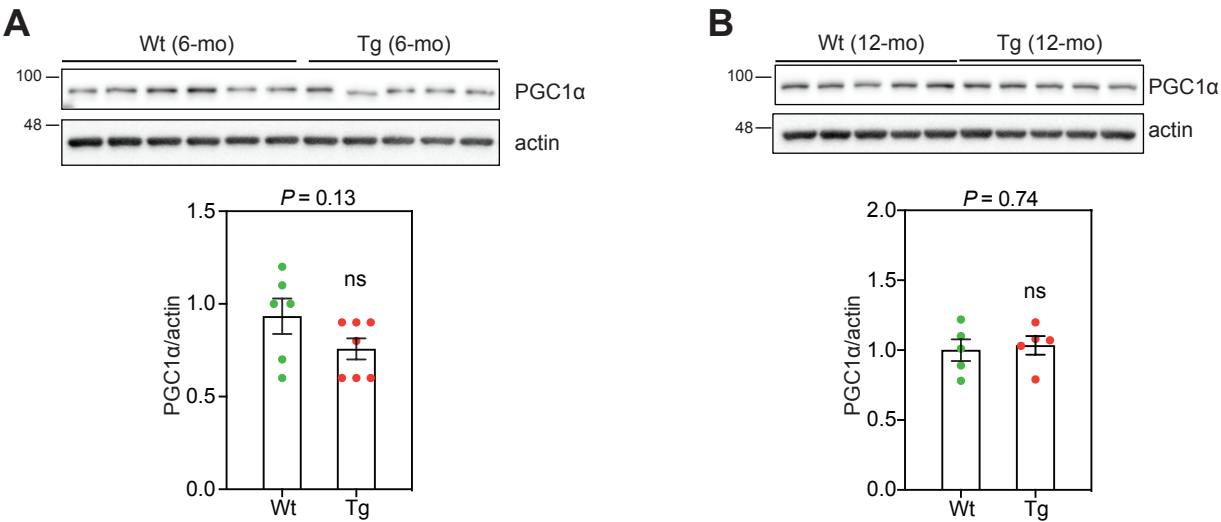

Supplement: Supplementary file 2 — Additional file 2 : Figure S2. Tau pathology does not affect the expression of the key regulator of mitochondrial biogenesis Pgc1α. WB analysis of extracts from 6-mo Wt and Tg mice CA1 (Wt, n = 6; Tg, n = 5) (A), and from 12-mo Wt and Tg mice hippocampi (Wt, n = 5; Tg, n = 6) (B) for PGC-1α. Actin was used as loading control. Data are presented as mean ± SEM. [file 40478_2020_896_MOESM2_ESM.pdf]

Figure S3

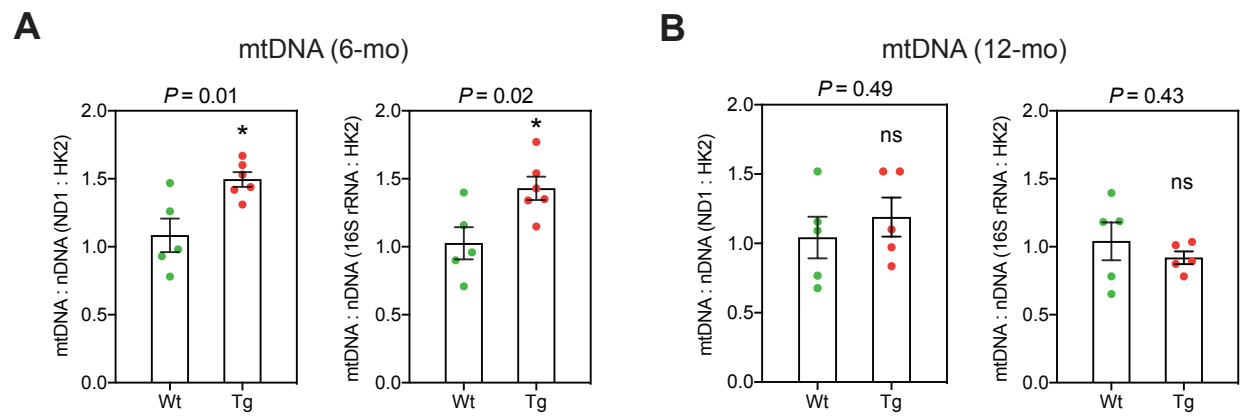

Supplement: Supplementary file 3 — Additional file 3 : Figure S3. Early tau pathology correlates with increased number of mtDNA molecules. (A) Quantitative-PCR analysis of mtDNA copy number was carried out in total DNA isolated from CA1 region from 6-mo Wt (n = 5) and Tg (n = 6) mice. Nd1 and 16 s rRNA are mitochondrial genes and Hk2 is a nuclear gene. (B) Analysis of mtDNA copy number in total DNA from 12-mo Wt (n = 5) and Tg (n = 5) mice hippocampi. Data are presented as mean ± SEM (*P < 0.05). [file 40478_2020_896_MOESM3_ESM.pdf]

Figure S4

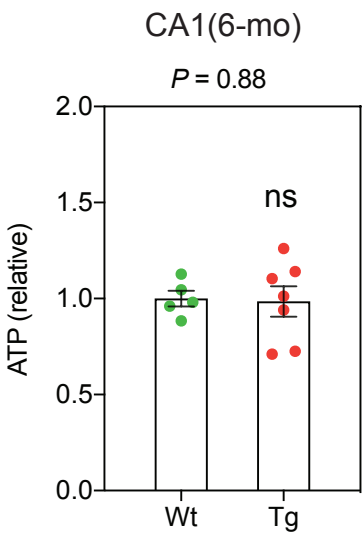

Supplement: Supplementary file 4 — Additional file 4 : Figure S4. ATP measurements in 6-mo Tg and Wt CA1. ATP was measured using a luciferase-based assay (ATPlite Luminescence Assay Kit, PerkinElmer), following the Manufacturer’s protocol. The content of ATP was normalized to protein content and presented as percentage of control. [file 40478_2020_896_MOESM4_ESM.pdf]

Figure S5

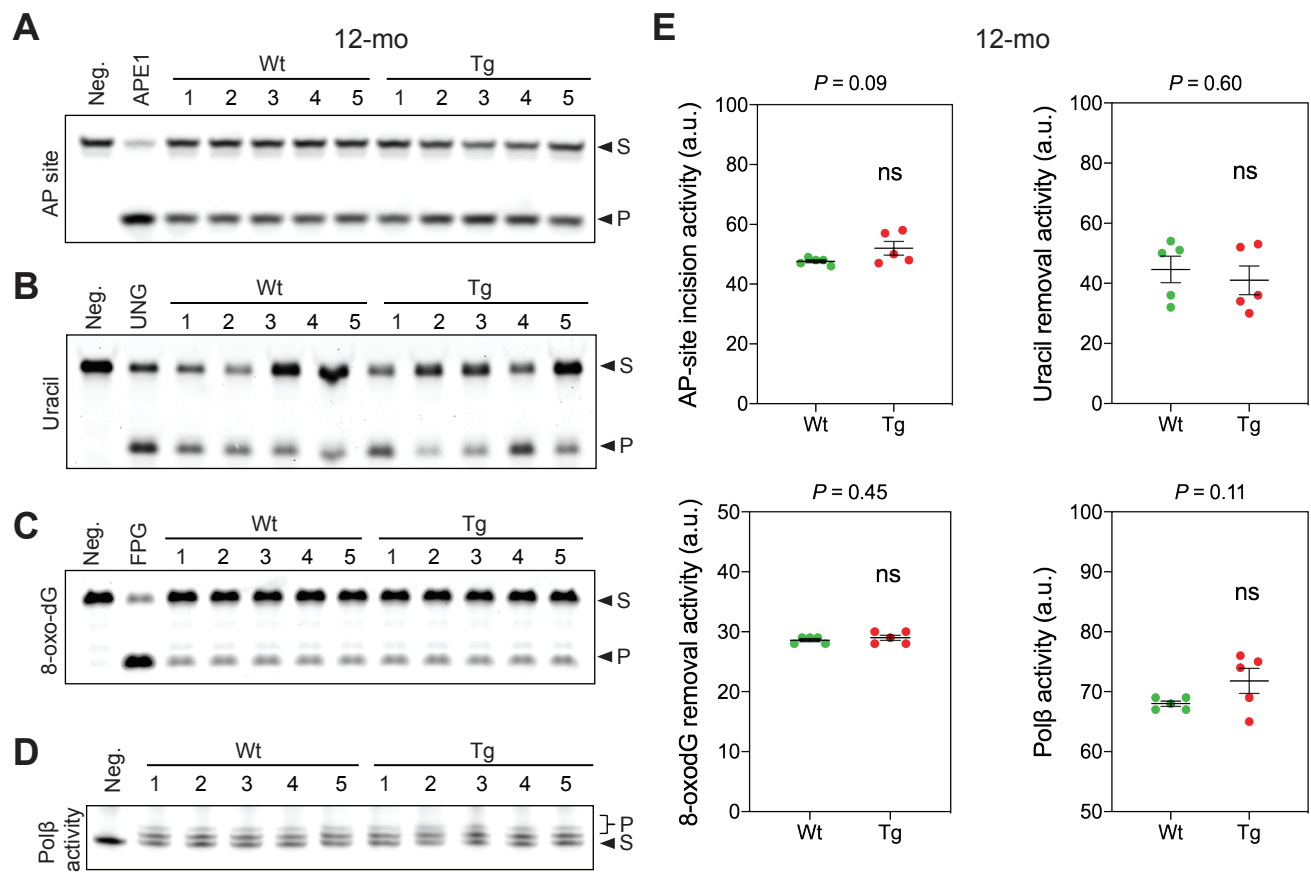

Supplement: Supplementary file 5 — Additional file 5 : Figure S5. BER activity is not changed in 12-mo Tg mice. Biochemical analysis of BER activity in hippocampal extracts from 12-mo Tg and Wt mice. (A) AP-site incision activity. Recombinant APE1 protein was used as a positive control. (B) Uracil removal activity. Purified recombinant UNG was used as a positive control. (C) 8-oxo-G removal activity Formamidopyrimidine DNA glycosylase (FPG) was used as a positive control. (D) Polβ nucleotide incorporation activity. (E) Quantifications of A-D, data are presented as mean ± SEM. [file 40478_2020_896_MOESM5_ESM.pdf]

Figure S6

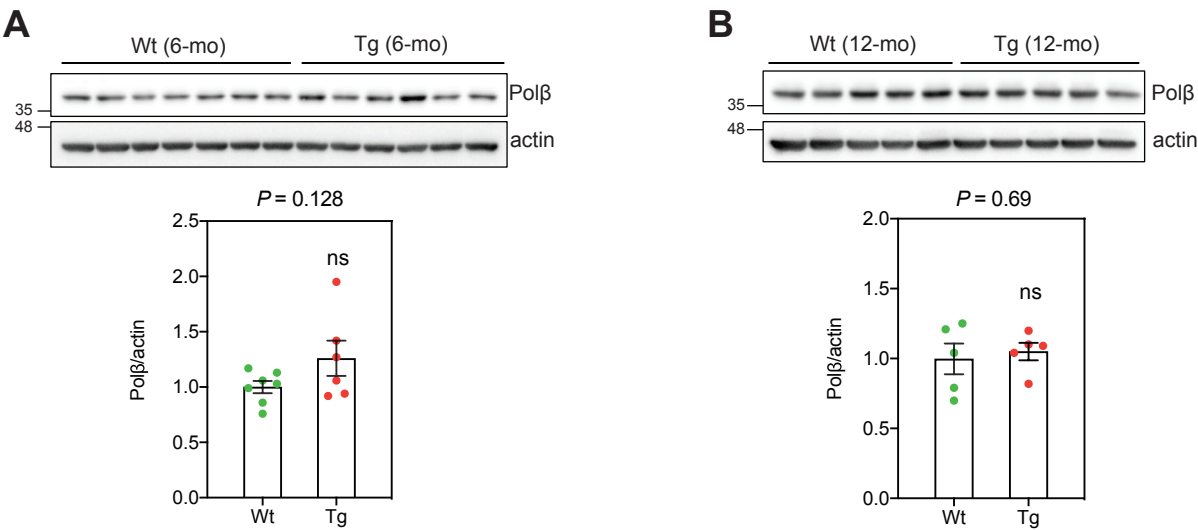

Supplement: Supplementary file 6 — Additional file 6 : Figure S6. Polβ protein level is not changed in Tg mice. WB analysis of extracts from 6-mo Wt and Tg mice CA1 (Wt, n = 7; Tg, n = 6) (A), and from 12-mo Wt and Tg mice hippocampi (Wt, n = 5; Tg, n = 5) (B) for Polβ. Actin was used as loading control. Data are presented as mean ± SEM. [file 40478_2020_896_MOESM6_ESM.pdf]

Figure S7

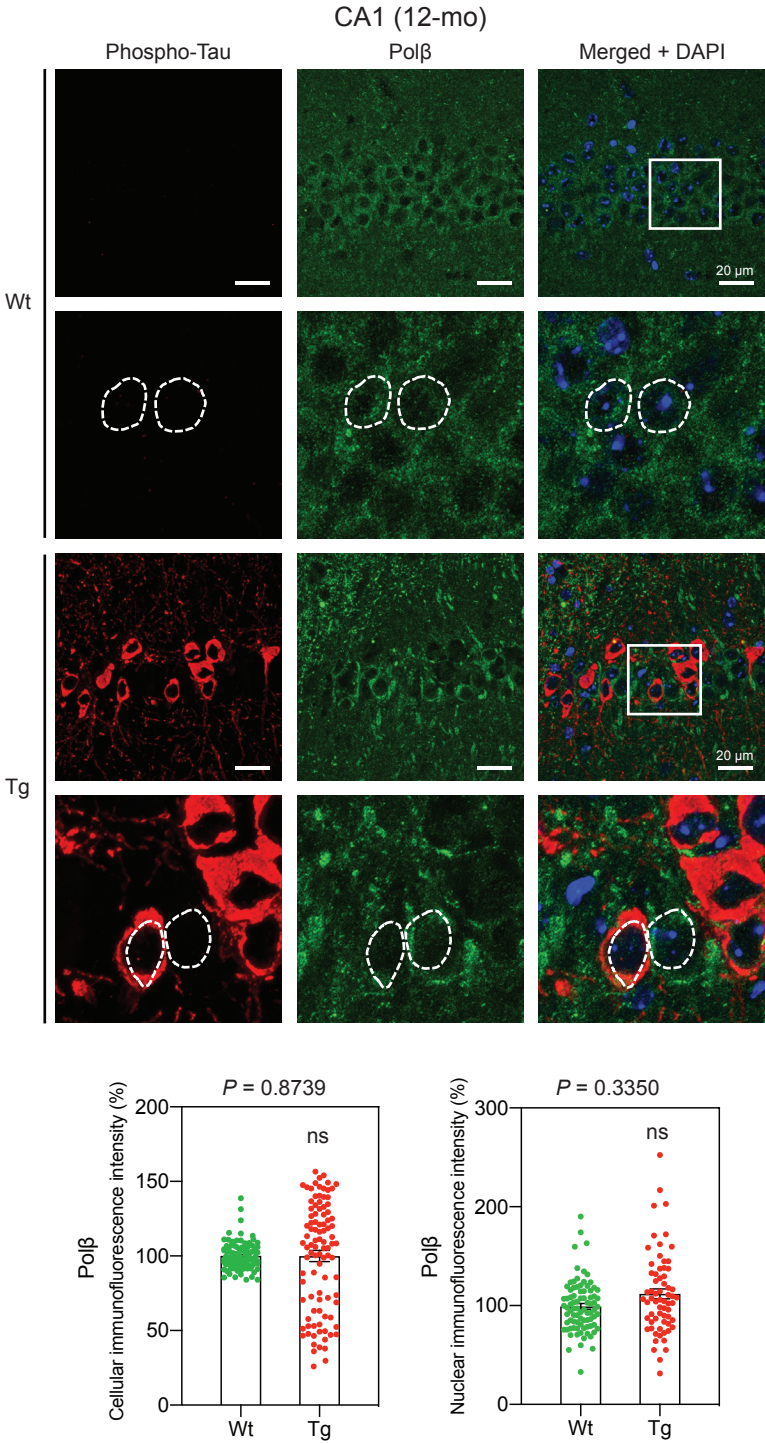

Supplement: Supplementary file 7 — Additional file 7 : Figure S7. Absence of cytoplasmic accumulation of Polβ in 12-mo Tg mice hippocampal neurons. Representative images of sagittal CA1 sections from 12-mo Wt and Tg mice hippocampi. The sections were co-labeled with anti-phospho-tau (AT8), and Polβ antibodies (n = 3 for each mouse category). Immunofluorescence signals were analyzed using laser scanning confocal microscopy (z projection). Nuclei were detected with DAPI staining. Representative nuclei are delimitated by white dashed lines. The scale bars represent 20 μm. The intensity of the cellular and nuclear Polβ fluorescence signals was quantified within CA1 cells from 12-mo Wt and Tg hippocampi (nuclei: Wt, n = 84; Tg, n = 66; cellular: Wt, n = 97; Tg, n = 98). Graph shows the mean of nuclear fluorescence per mouse category. Data are presented as mean ± SEM. [file 40478_2020_896_MOESM7_ESM.pdf]

Figure S8

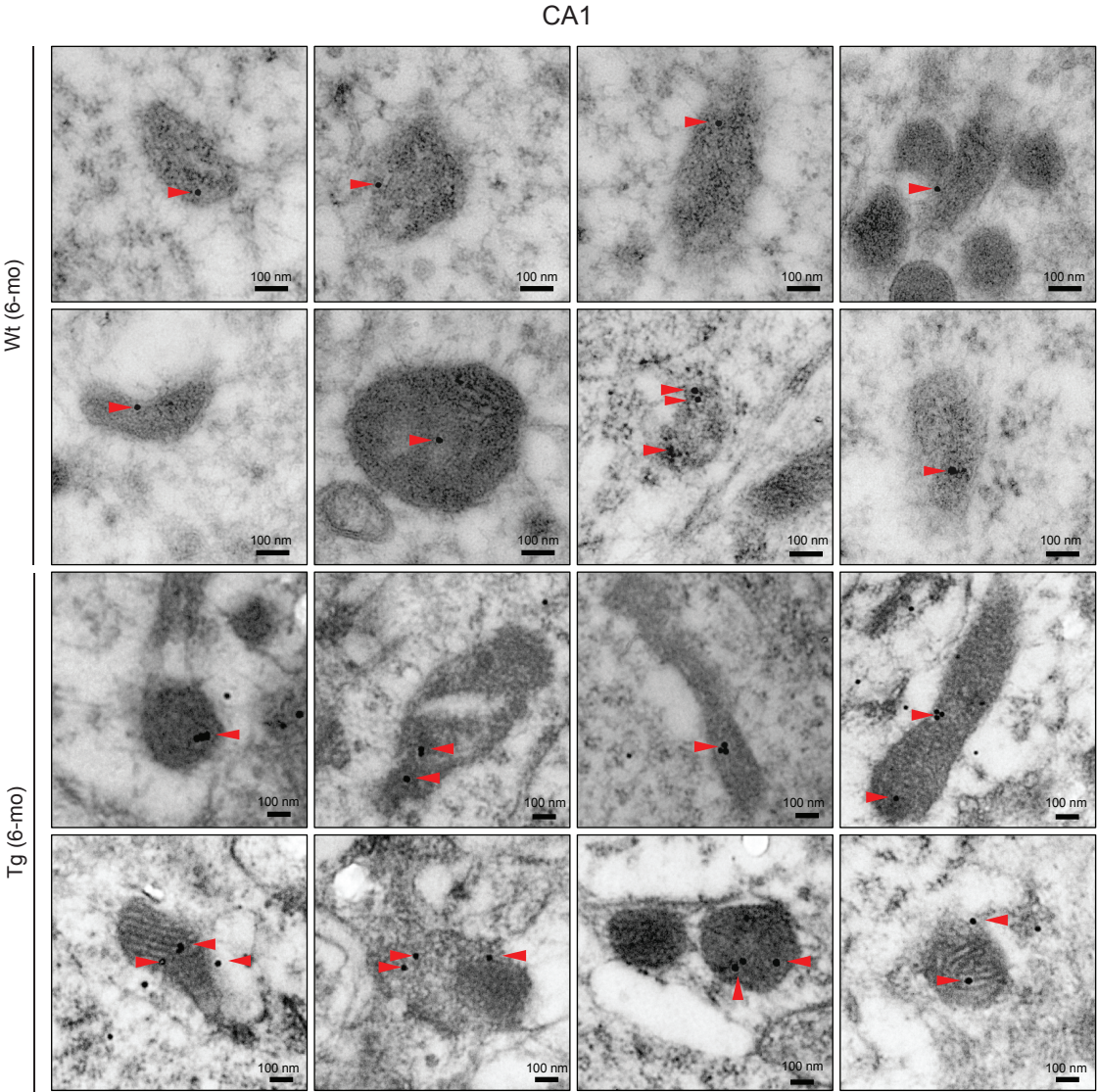

Supplement: Supplementary file 8 — Additional file 8 : Figure S8. Increased mitochondrial Polβ in Tg mice hippocampal neurons. Representative immunoelectron microscopy images of CA1 sections from 6-mo Tg and Wt mice hippocampus. The sections were labeled with Polβ antibodies (n = 3 for each mouse category). The scale bars represent 100 nm. Red arrows point Polβ localization [file 40478_2020_896_MOESM8_ESM.pdf]

Figure S9

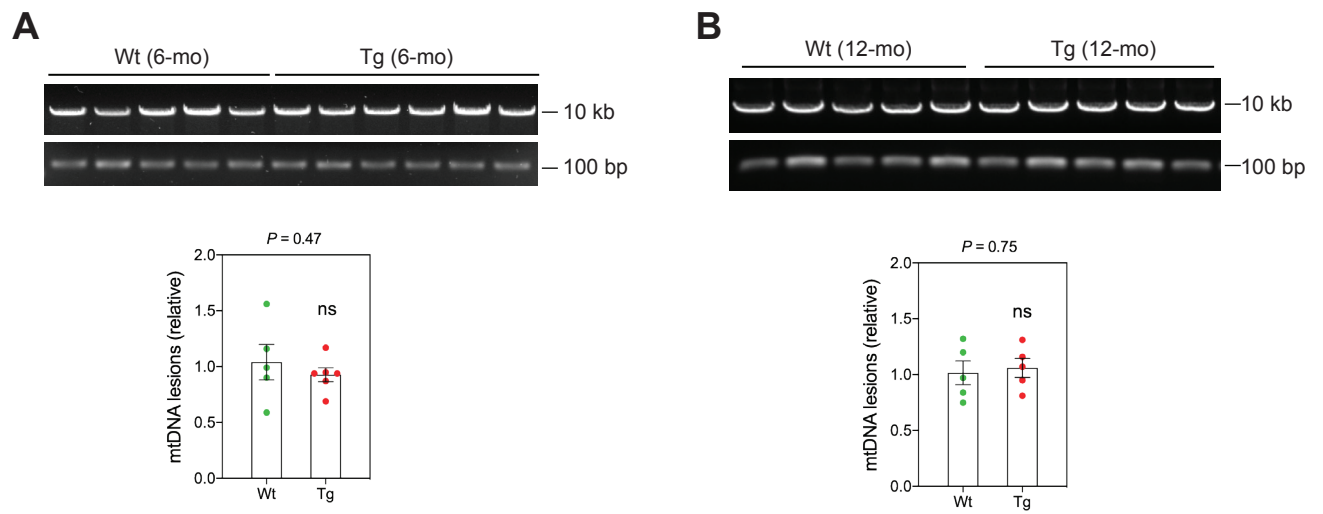

Supplement: Supplementary file 9 — Additional file 9 : Figure S9. PCR-based mtDNA damage analysis. (A) MtDNA damage analysis from 6-mo Wt (n = 5) and Tg (n = 6) mice CA1 region by long range PCR. (B) MtDNA analysis of 12-mo Wt (n = 5) and Tg (n = 5) mice hippocampi. Data are presented as mean ± SEM. Statistics were performed with unpaired two-tailed Mann-Whitney test (**P < 0.01) [file 40478_2020_896_MOESM9_ESM.pdf]

Figure S10

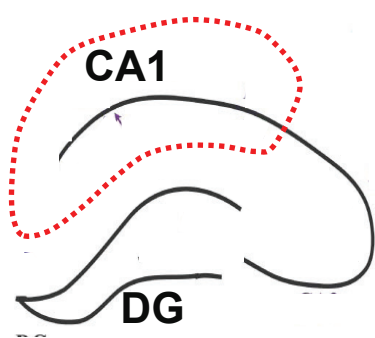

Supplement: Supplementary file 10 — Additional file 10 : Figure S10. Schematic representation of a coronal mouse hippocampal section. The dashed red line shows the dissected CA1 region. [file 40478_2020_896_MOESM10_ESM.pdf]

Figure S11

A

Frontal cortex AD

Polβ

Tau oligomers

Merged + DAPI

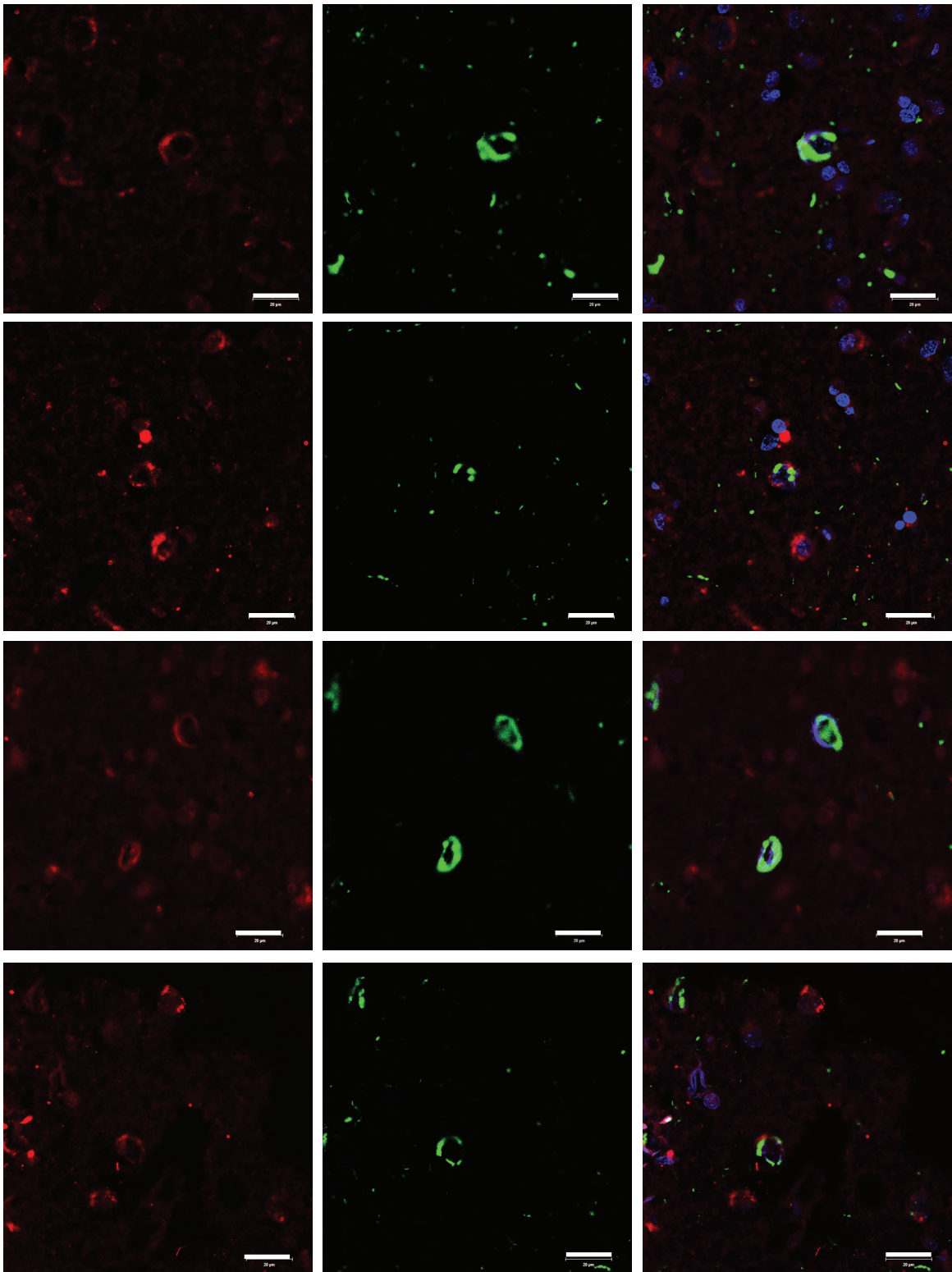

Figure S11

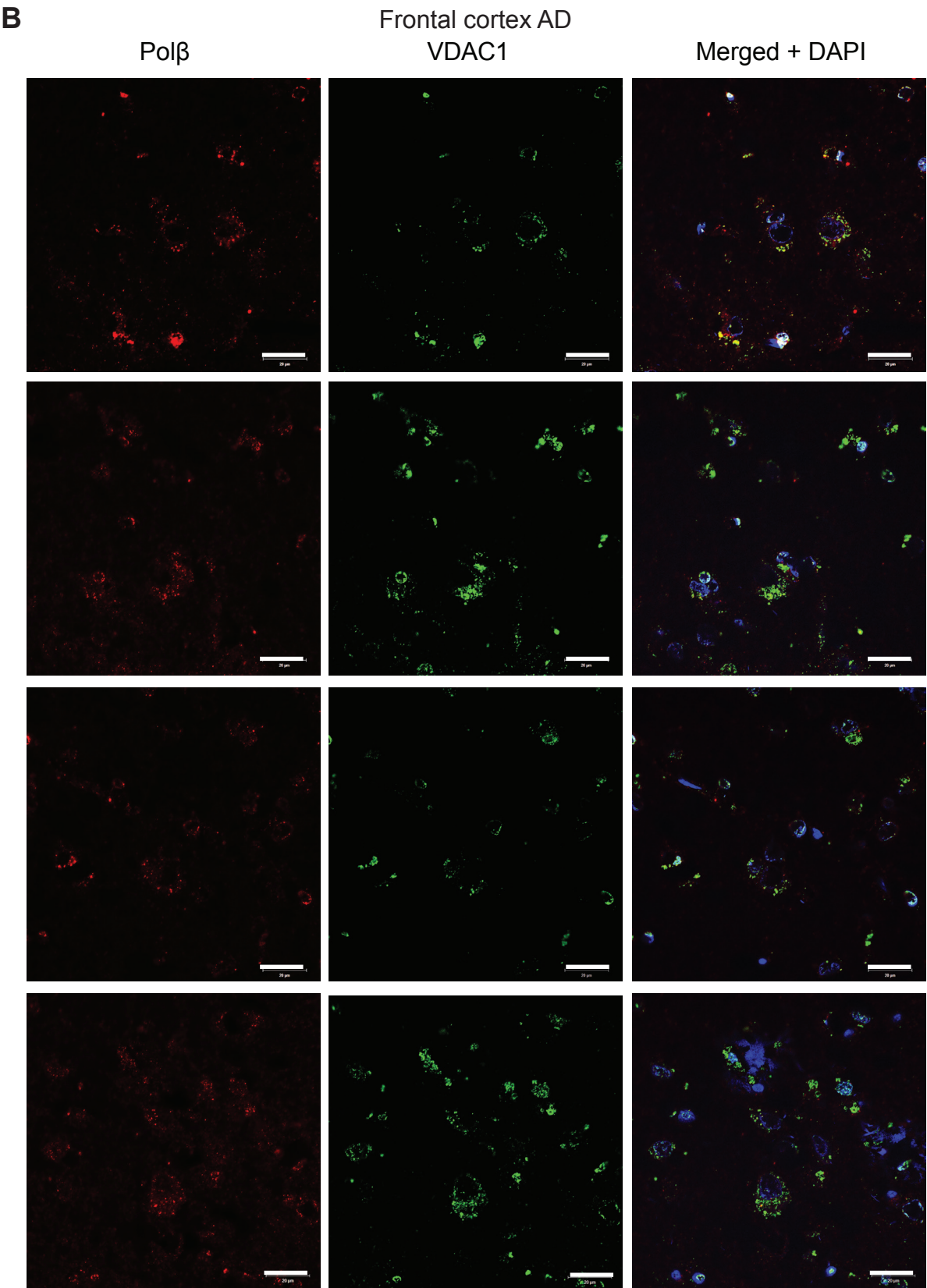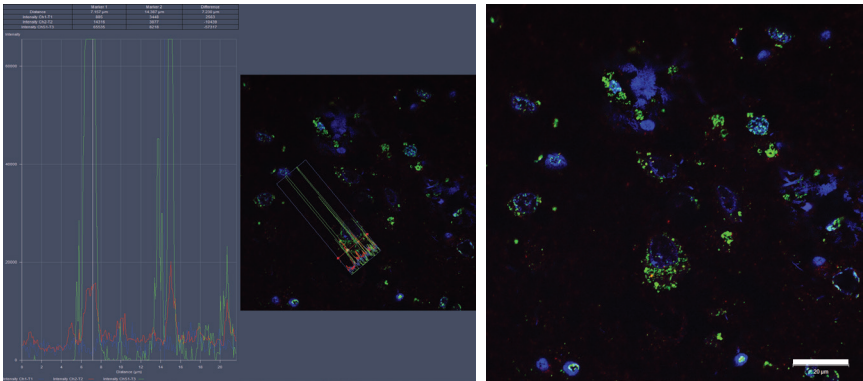

Supplement: Supplementary file 11 — Additional file 11 : Figure S11. Increased cytoplasmic and mitochondrial accumulation of PolB in neurons from AD brain. Representative images of frontal cortex sections from human control (Ctr) and Braak VI Alzheimer (AD) frontal cortex. Immunofluorescence signals were analyzed by laser scanning confocal microscopy. Nuclei were detected with DAPI staining. The scale bars represent 20 μm. (A) The sections were labeled with the tau oligomer antibody, TOC1, and anti-Polβ antibody (n = 3 for each category) (z projection) or (B) with antibodies against Polβ and VDAC1 (single confocale section). [file 40478_2020_896_MOESM11_ESM.pdf]
