# Supplementary material for: Hippocampal tau oligomerization early in tau pathology coincides with a transient alteration of mitochondrial homeostasis and DNA repair in a mouse model of tauopathy
Source: Acta Neuropathol Commun. 2020 Mar 4;8:25. doi: 10.1186/s40478-020-00896-8 (PMC7057491; doi:10.1186/s40478-020-00896-8)
Supplement: Supplementary file 12 — Additional file 12 : Figure S12. List of oligonucleotides used in mtDNA analysis and in BER assays. [file 40478_2020_896_MOESM12_ESM.pdf]

Figure S12

Primers used in mtDNA copy number analysis:
ND1 forward primer: 5´-CTAGCAGAAACAAACCGGGC-3´,
and ND1 reverse primer: 5´-CCGGCTGCGTATTCTACGTT-3´,
16S rRNA forward primer: 5´-CCGCAAGGGAAAGATGAAAGAC-3´,
and 16S rRNA reverse primer: 5´-TCGTTTGGTTTCGGGGTTTC-3´,
HK2 forward primer: 5´-GCCAGCCTCTCCTGATTT TAGTGT-3´,
and HK2 reverse primer: 5´-GGGAACACAAAAGACCTCTTCTGG-3´.
The CT values of ND1 and 16S rRNA were normalized to nuclear genomic DNA (HK2).

Primers used for mtDNA integrity analysis: forward primer; 5´-GCCAGCCTGACCCATAGCCATAATAT-3´,
and reverse primer; 5´-GAGAGATTTTATGGGTGTAATGCGG-3´, to amplify a 10 kb region of mtDNA.
And primers, forward; 5′- CCCAGCTACTACCATCATTCAAGT -3´, and reverse primer;
5′- GATGGTTTGGGAGATTGGTTGATGT -3′, were used to amplify a 100 bp fragment of mtDNA.

Oligonucleotides used in BER analysis. The following oligonucleotides were annealed to their complementary oligos for;
1- AP site incision assay (AP:G), 5´-TAMRA-GATCCTCTAGAGXCGACCTGCA-3´,
2- uracil-excision assay (U:G), 5´-TAMRA-GATCCTCTAGAGUCGACCTGCA-3´,
and 3- 8-oxoG-excision assay (8-oxoG:C) 5´-GAACGACTGT8-oxo-GACTTGACTGCTACTGAT-3´-TAMRA.
For the gap filling assay the following oligonucleotides were annealed to prepare DNA substrate with a single nucleotide gap:
5´-TAMRA-CATATCCGTGTCGCCCTC-3´, 5´-TTCCGATAGTGACTACA,
and 3´-GTATAGGCACAGCGGGAGTAAGGCTATCACTGATGT-5´.
